# Postnatal Fluoxetine Treatment Alters Perineuronal Net Formation and Maintenance in the Hippocampus

**DOI:** 10.1523/ENEURO.0424-20.2021

**Published:** 2021-04-05

**Authors:** Sourish Mukhopadhyay, Ashmita Chatterjee, Praachi Tiwari, Utkarsha Ghai, Vidita A. Vaidya

**Affiliations:** Department of Biological Sciences, Tata Institute of Fundamental Research, Mumbai 400005, India

**Keywords:** antidepressant, selective serotonin reuptake inhibitor, interneurons, parvalbumin, PNN

## Abstract

Elevation of serotonin via postnatal fluoxetine (PNFlx) treatment during critical temporal windows is hypothesized to perturb the development of limbic circuits thus establishing a substratum for persistent disruption of mood-related behavior. We examined the impact of PNFlx treatment on the formation and maintenance of perineuronal nets (PNNs), extracellular matrix (ECM) structures that deposit primarily around inhibitory interneurons, and mark the closure of critical period plasticity. PNFlx treatment evoked a significant decline in PNN number, with a robust reduction in PNNs deposited around parvalbumin (PV) interneurons, within the CA1 and CA3 hippocampal subfields at postnatal day (P)21 in Sprague Dawley rat pups. While the reduction in CA1 subfield PNN number was still observed in adulthood, we observed no change in colocalization of PV-positive interneurons with PNNs in the hippocampi of adult PNFlx animals. PNFlx treatment did not alter hippocampal PV, calretinin (CalR), or Reelin-positive neuron numbers in PNFlx animals at P21 or in adulthood. We did observe a small, but significant increase in somatostatin (SST)-positive interneurons in the DG subfield of PNFlx-treated animals in adulthood. This was accompanied by altered GABA-A receptor subunit composition, increased dendritic complexity of apical dendrites of CA1 pyramidal neurons, and enhanced neuronal activation revealed by increased c-Fos-positive cell numbers within hippocampi of PNFlx-treated animals in adulthood. These results indicate that PNFlx treatment alters the formation of PNNs within the hippocampus, raising the possibility of a disruption of excitation-inhibition (E/I) balance within this key limbic brain region.

## Significance Statement

Clinical and preclinical studies indicate that developmental exposure to fluoxetine programs persistent dysregulation of mood-related behaviors. This is hypothesized to involve the disruption of key brain regions, such as the hippocampus that regulate mood behaviors. We show that postnatal exposure to fluoxetine alters hippocampal perineuronal nets (PNNs), extracellular matrix (ECM) structures that regulate plasticity. The decline in PNNs is noted in early postnatal life, and persists into adulthood in specific hippocampal subfields. Adult animals with a history of postnatal fluoxetine (PNFlx) exposure exhibit altered numbers of somatostatin (SST) interneurons, GABA receptor subunit expression and neuronal activation within the hippocampus. Collectively our findings indicate that PNFlx treatment exerts both acute and persistent effects on hippocampal structure and neuronal activity.

## Introduction

Selective serotonin reuptake inhibitors (SSRIs) are conventionally used as the first line of treatment for women with gestational and postpartum depression (Nonacs and Cohen, 2003; Kodish et al., 2011). Perinatal exposure to SSRIs has been linked to altered neurobehavioral development (Mulder et al., 2011; Glover and Clinton, 2016) and increased risk for suicidal ideation in children and adolescents (Cipriani et al., 2005). Preclinical studies investigating perinatal exposure to SSRIs report increased anxiety and despair-like behaviors in rodent models that persist across the life-span (Hilakivi and Hilakivi, 1987; Ansorge et al., 2004, 2008; Sarkar et al., 2014a). In addition to altering the development of emotionality, perinatal SSRI administration also evokes dysregulation of circadian rhythms and sleep (Mirmiran et al., 1981; Hilakivi et al., 1988; Vogel et al., 1990; Popa et al., 2008), alters cortical network function (Simpson et al., 2011), perturbs hormonal stress responses (Norcross et al., 2008), disrupts social play (Bond et al., 2020), reproductive and maternal care behavior (Svirsky et al., 2016), and alters the development of serotonergic circuitry (Weaver et al., 2010). Perinatal SSRI exposure alters hippocampal plasticity (Karpova et al., 2009), neurotrophic factor signaling (Rayen et al., 2015), and disrupts hippocampal neurogenesis in a sexually dimorphic manner (Gemmel et al., 2016). Given the extended developmental trajectory of the hippocampus and the key role it plays in emotional behavior (Rice and Barone, 2000), this limbic brain region that receives dense serotonergic innervation (Oleskevich and Descarries, 1990) is likely to be impacted by perinatal SSRI administration.

The hippocampus exhibits protracted development with neurogenesis, gliogenesis, cell-type specification, and synaptogenesis extending well into the juvenile window for rodents (Bayer, 1980). Preclinical models of early life stress are reported to evoke a decline in hippocampal neurogenesis (Naninck et al., 2015), dendritic atrophy (Champagne et al., 2008), enhanced cell death (Bath et al., 2016), and altered neurotrophic signaling in the hippocampus (Suri et al., 2014). These structural and functional changes in the hippocampus are implicated in mediating the sustained changes in mood-related behavior that arise as a consequence of early adversity (Fenoglio et al., 2006; Chen and Baram, 2016). The postnatal window marks an important period in hippocampal development and closure of this period is associated with the deposition of extracellular matrix (ECM) moieties, called perineuronal nets (PNNs), preferentially deposited around interneurons (Hensch, 2005). Maternal separation (MS)-based models of early stress are reported to cause a decline of PNNs in the prelimbic (PrL) prefrontal cortex (PFC) in juvenile animals (Gildawie et al., 2020), and an increase in PNN intensity around parvalbumin (PV) interneurons in the hippocampus in adults (Murthy et al., 2019). Chronic administration of SSRIs can also impact PNN deposition around PV cells, and is implicated in fear erasure in the basolateral amygdala (Karpova et al., 2011) and in the reopening of critical period plasticity in the visual cortex, likely through the dissolution of PNNs in adulthood (Maya Vetencourt et al., 2008). We hypothesized that SSRI administration during postnatal life could impact the formation and maintenance of PNNs in the hippocampus, a brain region reported to be highly sensitive to perturbations of serotonergic signaling.

In this study, we investigated the influence of postnatal fluoxetine (PNFlx) treatment on the formation of PNNs during postnatal life and their maintenance in adulthood. We addressed whether the number of PNNs that encapsulate PV-positive interneurons in distinct hippocampal subfields are altered as a consequence of PNFlx treatment, both at an early time point in postnatal life soon after cessation of the SSRI treatment and in adulthood. PNNs are known for the role they play in regulating synaptic physiology and receptor composition, and can thus impinge on neuronal excitation-inhibition (E/I) balance (Sorg et al., 2016; Fawcett et al., 2019). We also examined the influence of PNFlx treatment on interneuron numbers, NMDA and GABA receptor subunit composition, neuronal activity in the hippocampus and the cytoarchitecture of hippocampal pyramidal neurons. We find that PNFlx treatment results in reduced numbers of PNNs in the hippocampus and that this decline is maintained into adulthood in a hippocampal subfield-specific manner. This reduction in PNNs results in a significant decline in the number of PV cells ensheathed by PNNs. Additionally, we also observed an increase in neuronal activity within the hippocampus in animals with a history of PNFlx, associated with an altered subunit composition in GABA-A receptors, and a small but significant increase in the dendritic complexity noted in the distal regions of CA1 pyramidal neuron dendritic arbors. Taken together, our results indicate that chronic exposure to SSRIs in the postnatal window alters the trajectory of PNN development, and has implications for the function of local inhibitory circuits in the hippocampus.

## Materials and Methods

### Animals

Sprague Dawley rats bred at the Tata Institute of Fundamental Research (TIFR) animal facility were used for all experiments. Animals were maintained on a 12/12 h light/dark cycle (7 A.M. to 7 P.M.) with *ad libitum* access to food and water. Experimental procedures were conducted as per the guidelines of the Committee for the Purpose of Control and Supervision of Experiments on Animals (CPCSEA), Government of India and were approved by the TIFR animal ethics committee (TIFR/IAEC/2017-2).

### Drug treatments

Litters derived from primiparous Sprague Dawley dams were assigned at random to either the vehicle or PNFlx administered treatment groups, with each treatment group containing pups obtained from four or more litters, to avoid any litter-specific effects. Rat pups received oral administration of fluoxetine (10 mg/kg; kind gift from IPCA Laboratories) or vehicle (5% sucrose, Sigma-Aldrich) once daily through a micropipette (0.5–10 μl, Eppendorf) from postnatal day (P)2 to P21, completed within 3 min of separation from the dam to minimize any handling related stress. Male rat pups were weaned between P25 and P30, following which they were housed in identical group-housing conditions (three to four animals per cage).

### Immunohistochemistry

Sprague Dawley male and female rat pups were killed at P21 and adult male rats were killed at P80 via transcardial perfusion with 4% paraformaldehyde. Harvested brains were sectioned on a VT1000S vibratome (Leica) to generate serial coronal sections (40-μm thickness). Six free floating sections, containing the hippocampus and the medial PFC (mPFC; 240 μm apart), per animal were processed for each immunohistochemical staining. Sections were blocked in 10% horse serum made in 0.1 m phosphate buffer with 0.3% Triton X-100 (PBTx) for 2 h followed by overnight incubation with the following primary antibodies: mouse anti-PV (1:1000, Sigma-Aldrich, P3088), goat anti-somatostatin (SST; 1:350, Santa Cruz, SC-7819), goat anti-calretinin (CalR; 1:1000, Santa Cruz, SC-26512), mouse anti-Reelin (1:1000, Sigma-Aldrich, MAB5364), and rabbit anti-c-Fos (1:1000, Cell Signaling Technology, #2250) in 0.3% PBTx, at room temperature. After washes, the sections were incubated with secondary antibody solutions of biotinylated horse anti-mouse (1:500, Vector Laboratories, BA2000), biotinylated donkey anti-rabbit (1:500, Millipore, AP182B), and biotinylated rabbit anti-goat (1:500, Millipore, AP106) in 0.3% PBTx for 3 h. Subsequently, sections were incubated with avidin-biotin complex (Vector Labs, PK-6100) in 0.1 m PB for 1.5 h and visualized with diaminobenzidine staining (Sigma-Aldrich, D4293). To detect PNNs, sections were incubated with the plant lectin, biotinylated *Wisteria floribunda* (WFA; 1:250, Vector Laboratories, B1355) in 0.3% PBTx overnight, followed by incubation with secondary antibody solution of Alexa Fluor 488-conjugated donkey anti-streptavidin (1:500, Invitrogen, S11223) for 3 h. For double immunostainings of PV and PNN, sections were incubated with primary antibody solution of rabbit anti-PV (1:1000) and biotinylated WFA (1:250) in 0.3% PBTx overnight followed by incubation with secondary antibody solutions of Alexa Fluor 488-conjugated donkey anti-streptavidin (1:500) and Alexa Fluor 555-conjugated donkey anti-rabbit (1:500, Invitrogen, A31572) for 3 h.

### Cell visualization and counting

Immunostained sections were mounted on glass slides with DPX mountant medium (Merck, 100579). Slides were coded and quantification was conducted by the experimenter blind to the treatment groups. Cells were visualized at 20× magnification using a bright field microscope (Zeiss Axioscope 2). For cell counting analysis in the subfields of the hippocampus, total number of stained cells were counted per animal per hippocampal subfield, namely the CA1, CA3, and DG, across representative sections taken from both the dorsal and ventral hippocampus, and divided by the number of sections to obtain an average number of cells per section within the respective hippocampal subfield. For cell counting analysis in the subdivisions of the mPFC, cells were visualized at 10× magnification in the infralimbic (IL), PrL, and cingulate (Cg) subdivisions, and images were taken using a Zeiss Axiocam camera. Cell numbers were counted manually using ImageJ (National Institutes of Health) by an experimenter blinded to the treatment conditions. For PV and PNN double immunofluorescence, sections spanning the rostro-caudal axis of the hippocampus from each animal were mounted on glass slides with Vectashield Hard-set Antifade mounting media with DAPI (Vector Labs, H-1500). Slides were coded and quantification was conducted by the experimenter blind to the treatment groups. Cells were visualized at 20× magnification and images were acquired using Zeiss Axio Imager M2 (Zeiss). The percent colocalization of PV-positive cells with the PNN marker WFA was determined in the DG, CA1, CA3 hippocampal subfield in six sections (240 μm apart) per animal. A minimum of 50 PV-positive cells were analyzed per animal using *z*-plane sectioning (0.5 μm) to confirm the percent colocalization of PV with the PNN marker WFA.

### Quantitative PCR (qPCR)

Vehicle and PNFlx treatment groups were subjected to qPCR analysis at two time points, namely P21, 2 h after cessation of the final fluoxetine/vehicle treatment and in adulthood (P100). The hippocampus was micro-dissected, snap-frozen using liquid nitrogen, and stored at −80°C. RNA was extracted using TRIzol reagent (Thermo Fisher Scientific) and reverse transcribed using cDNA synthesis kit (PrimeScript first strand cDNA Synthesis kit, Takara Bio). The synthesized cDNA was subjected to qPCR using KAPA SYBR (KAPA Biosystems) and a Bio-Rad CFX96 real-time PCR machine. Primers were designed using the NCBI primer BLAST program. The complete list of primers used is provided in Table 1. Hypoxanthine phosphoribosyltransferase 1 (*Hprt-1*) was used as the housekeeping gene for normalization.

**Table 1:** Primer sequences (5’−3’)

Gene	Forward primer sequence	Reverse primer sequence
*aggrecan*	CCTCAGAGGTGAATGTTACCG	TGGAGAAGCAAGGGTAGGG
*neurocan*	CCTTCCTCCCTCTCAATTCC	CCAAGACCAAAGACCAGAGC
*brevican*	CTTTCCCCGAGTCAAATGG	TAGACCCCGGAATCATTGG
*versican*	CAACCTTGCCCACCTTACC	TGCGTAGGCACTGATACCC
*hapln1*	ACCAGGATGCTGTGATTGC	TCCCAGAACCCGTAGTTCC
*hapln2*	GCGTGCCTATCAACTGTGC	TGAATGACCTCGTGGATGG
*cspg4*	CTGGAGAGAGGTGGAAGAGCAG	AACAGGGAGGATGGTGATAGTG
*tenascin c*	AAATGCAGCCACAGTCAGC	CATGGCAATCACACTGACG
*tenascin r*	ACAGGCCAAACCTCAGACC	TGACAGCAGTTGGTGTTGC
*has1*	GTGCTCACGATCACCTTCG	TTCGGTGCTCCAGGTAAGC
*has2*	GCAGGAGCTGAACAAGATGC	TTGGATGATGAGGTGTGAGG
*has3*	GATCGGCACCTTACCAACC	CCACAGGTGGTGCTTATGG
*chst3*	TCGCTTTGCCTCAGGATTGC	GCTTGTCGGAGACCCTGGATA
*chst7*	CCCGGGGCATATCTAGGTCA	CTGTGCAGCCTCTTCAGTGT
*chst11*	CAGAATTTGCCGGATGGTGC	GGATTCCTCCGCATGACTGG
*chst12*	GGCCACACATCCTAGAACCC	GCGTCAGAACTAAGGAGCGT
*chst13*	ATGGGAAGGCGCTCCTGTTG	TGTTTTCAAATGCGGGACGC
*timp1*	CTTCCTGGTTCCCTGGCATA	ATCGCTCTGGTAGCCCTTCT
*timp4*	ACTCTTCTCTCTGTGGTGTGA	GCATAGCAAGTGGTGATTTGGC
*mmp2*	CCAGAGACTGCTATGTCCACT	ACACCACACCTTGCCATCG
*mmp9*	CATCTGTATGGTCGTGGCTCT	CTGTCGGCTGTGGTTCAG

### Western botting

Sprague Dawley male and female pups were killed at P21 and adult male Sprague Dawley rats were killed on P100 and their hippocampi were dissected, snap-frozen in liquid nitrogen, and stored at −80°C. Tissue homogenization and protein extraction were performed in radioimmunoprecipitation assay buffer [10 mm Tris-Cl (pH 8.0), 1 mm EDTA, 0.5 mm EGTA, 1% Triton X-100, 0.1% sodium deoxycholate, 0.1% SDS, 140 mm NaCl]. Protease inhibitor cocktail (Sigma-Aldrich, P8340), phosphatase inhibitor cocktail 2 (Sigma-Aldrich, P5276), and phosphatase inhibitor cocktail 3 (Sigma-Aldrich, P0044) were added to the extraction buffer before protein extraction. Protein concentrations were estimated using a Quantipro BCA assay kit (Sigma-Aldrich). Protein lysates (50 μg) were resolved on an SDS-PAGE system and transferred onto polyvinylidene fluoride membranes. The membranes were blocked using 5% bovine serum albumin (BSA; Sigma-Aldrich, A9418) in Tris-buffered saline with 0.1% of Tween 20 (TBST) followed by incubation with the primary antibodies, namely rabbit anti-NR2A (1:1000, Millipore, 07-632), rabbit anti-NR2B (1:1000, Millipore, 06-600), rabbit anti-GABA-Aɑ1 (1:1000, Abcam, ab33299), mouse anti-GABA-Aɑ2 (1:1000, Abcam, ab193311), and rabbit anti-β-Actin (1:10,000, ABclonal, AC026) in TBST with 5% BSA overnight at 4°C. Following washes the blots were incubated with horseradish peroxidase (HRP)-conjugated goat anti-rabbit (1:5000, ABclonal, AS014) or HRP-conjugated goat anti-mouse (1:5000, ABclonal, AS003) for 2 h. Visualization of the signal was performed with a Western blotting detection kit (SuperSignal West Pico Plus, Thermofisher, 34579) using the GE GE HealthcareImager 600 (GE Life Sciences). Densitometric analysis of the blots were performed using ImageJ.

### Golgi staining and arborization analysis

Male Sprague Dawley rats were killed in adulthood at P120 and their brains were harvested and Golgi staining was conducted using the FD Rapid GolgiStain kit (PK401) as per the manufacturer’s instructions. Each brain was cut coronally into a smaller chunk containing the entire hippocampus and incubated with the impregnation solution in the dark for 21 d. After impregnation, the brains are placed in a staining solution for 3 d. Sections (150 μm) were cut on a vibratome (Leica) and incubated with freshly prepared staining solutions as per the instructions in the FD Rapid GolgiStain kit for 10 min. After washes, the sections on slides were dehydrated using xylene, and mounted with DPX mountant medium. For tracing of neurons, slides were coded and quantification was conducted by an experimenter blind to the treatment groups. Tracing of CA1 pyramidal neurons was conducted at 20× magnification on the BX53 light microscope (Olympus) using the Neurolucida 10 (MBF Biosciences). For Sholl analysis of neuronal traces, the Neurolucida 10 Explorer (MBF Biosciences) was used.

### Statistical analysis

All experiments had two treatment groups and were subjected to the Shapiro-Wilk test to determine normality of the data. Data that followed a normal distribution were subjected to a two-tailed, unpaired Student’s *t* test using GraphPad Prism (GraphPad Software Inc.), and Welch’s correction was applied when variances varied significantly between the treatment groups. Data that did not exhibit a normal distribution were subjected to the Mann–Whitney *U* test. Graphs were plotted using GraphPad Prism. Data are expressed as mean ± SEM, and statistical significance was set at *p *<* *0.05. For the arborization analysis, Friedman’s test of repeated measures for non-parametric distributions was performed for Sholl analysis, and results were considered significant at *p *<* *0.05. A detailed statistical summary for all figures is provided in Tables 2, 3, and boldface in the table indicates *p *<* *0.05.

**Table 2:** Statistical table (except for **Fig. 3)**

Figure number	Figure detail	Data structure	Type of test	Confidence interval	*p* value
1	*B*	Normal	Student’s *t* test	21.30 to 53.30	**0.0003**
	*C*	Normal	Student’s *t* test	4.40 to 15.40	**0.0023**
	*D*	Normal	Student’s *t* test	−4.73 to 1.23	0.2002
	*F*, PV^+^ WFA^−^	Non-normal	Mann–Whitney *U* test	−0.46 to −0.05	**0.0286**
	*F*, PV^+^ WFA^+^	Non-normal	Mann–Whitney *U* test	0.05 to 0.46	**0.0286**
	*G*, PV^+^ WFA^−^	Non-normal	Mann–Whitney *U* test	−0.43 to −0.09	**0.0286**
	*G*, PV^+^ WFA^+^	Non-normal	Mann–Whitney *U* test	0.09 to 0.43	**0.0286**
	*H*, PV^+^ WFA^−^	Normal	Student’s *t* test	−0.13 to 0.03	0.1990
	*H*, PV^+^ WFA^+^	Normal	Student’s *t* test	−0.03 to 0.13	0.1990
2	*B*	Normal	Student’s *t* test	−23.71 to −0.78	**0.0379**
	*C*	Normal	Student’s *t* test	−9.31 to 0.79	0.0922
	*D*	Normal	Student’s *t* test	−5.38 to 4.70	0.8863
	*F*, PV^+^ WFA^−^	Normal	Student’s *t* test	−0.08 to 0.05	0.6638
	*F*, PV^+^ WFA^+^	Normal	Student’s *t* test	−0.05 to 0.08	0.6638
	*G*, PV^+^ WFA^−^	Normal	Student’s *t* test	−0.14 to 0.10	0.5950
	*G*, PV^+^ WFA^+^	Normal	Student’s *t* test	−0.10 to 0.14	0.5950
	*H*, PV^+^ WFA^−^	Non-normal	Mann–Whitney *U* test	−0.10 to 0.16	0.6870
	*H*, PV^+^ WFA^+^	Non-normal	Mann–Whitney *U* test	−0.16 to 0.10	0.6870
4	*C*, CA1	Normal	Student’s *t* test	−85.40 to 24.43	0.2306
	*C*, CA3	Normal	Student’s *t* test	−63.14 to 12.67	0.1594
	*C*, DG	Normal	Student’s *t* test	−16.70 to 12.98	0.7698
	*D*, CA1	Normal	Student’s *t* test	−40.69 to 24.94	0.5785
	*D*, CA3	Normal	Student’s *t* test	−10.45 to 23.48	0.3836
	*D*, DG	Normal	Student’s *t* test	−7.07 to −3.43	**0.0007**
	*F*, CA1	Normal	Student’s *t* test	−21.39 to 23.18	0.9284
	*F*, CA3	Normal	Student’s *t* test	−12.14 to 36.55	0.2811
	*F*, DG	Non-normal	Mann–Whitney *U* test	−15.03 to 20.72	0.4206
	*G*, CA1	Non-normal	Mann–Whitney *U* test	−32.39 to 98.72	0.6623
	*G*, CA3	Normal	Student’s *t* test	−67.94 to 42.92	0.6220
	*G*, DG	Non-normal	Mann–Whitney *U* test	−31.82 to 7.71	0.2468
	*I*, CA1	Normal	Student’s *t* test	−78.79 to 33.99	0.3321
	*I*, CA3	Normal	Student’s *t* test	−26.61 to 3.01	0.0914
	*I*, DG	Normal	Student’s *t* test	−10.22 to 20.88	0.3950
	*J*, CA1	Normal	Student’s *t* test	−34.27 to 6.37	0.1549
	*J*, CA3	Normal	Student’s *t* test	−10.45 to 23.48	0.6372
	*J*, DG	Normal	Student’s *t* test	−17.16 to 14.47	0.8533
	*L*, CA1	Normal	Student’s *t* test	−79.33 to 41.76	0.5022
	*L*, CA3	Normal	Student’s *t* test	−14.10 to 19.65	0.7234
	*L*, DG	Normal	Student’s *t* test	−31.34 to 24.28	0.7818
	*M*, CA1	Normal	Student’s *t* test	−54.22 to 10.70	0.1725
	*M*, CA3	Normal	Student’s *t* test	−14.59 to 3.37	0.2017
	*M*, DG	Normal	Student’s *t* test	−10.77 to 8.72	0.8242
5	*C*, NR2A	Normal	Student’s *t* test	−0.38 to 0.08	0.1694
	*C*, NR2B	Normal	Student’s *t* test	−0.71 to 0.05	0.0781
	*E*, GABA-Aα1	Normal	Student’s *t* test	−0.49 to 0.23	0.4172
	*E*, GABA-Aα2	Normal	Student’s *t* test	−0.60 to 0.61	0.9860
	*G*, NR2A	Normal	Student’s *t* test	−0.83 to 1.50	0.4880
	*G*, NR2B	Normal	Student’s *t* test	−1.14 to 1.03	0.9083
	*I*, GABA-Aα1	Normal	Student’s *t* test	−0.06 to 0.94	0.0723
	*I*, GABA-Aα2	Normal	Student’s *t* test	−0.83 to −0.05	**0.0330**
	*K*, CA1	Non-normal	Mann–Whitney *U* test	2.095 to 27.55	**0.0175**
	*K*, CA3	Non-normal	Mann–Whitney *U* test	1.60 to 8.69	**0.0105**
	*K*, DG	Normal	Student’s *t* test	−46.91 to −5.60	**0.0170**
	*M*	Non-normal	Friedman’s test		**<0.0001**
	*N*	Non-normal	Friedman’s test		**<0.0001**
6	*B*, IL	Normal	Student’s *t* test	−12.65 to 24.94	0.4543
	*B*, PrL	Non-normal	Mann–Whitney *U* test	−15.08 to 85.25	0.3333
	*B*, Cg	Normal	Student’s *t* test	−19.61 to 30.11	0.6329
	*C*, IL	Normal	Student’s *t* test	−33.70 to 3.95	0.1060
	*C*, PrL	Normal	Student’s *t* test	−34.51 to −3.62	**0.0216**
	*C*, Cg	Normal	Student’s *t* test	−25.93 to 3.48	0.1165
	*D*, IL	Normal	Student’s *t* test	−11.66 to 20.18	0.5470
	*D*, PrL	Normal	Student’s *t* test	−30.25 to 10.09	0.2825
	*D*, Cg	Normal	Student’s *t* test	−24.61 to 37.61	0.6428
	*E*, IL	Normal	Student’s *t* test	−14.26 to 14.24	0.9986
	*E*, PrL	Normal	Student’s *t* test	−2.37 to 24.83	0.0946
	*E*, Cg	Normal	Student’s *t* test	−22.92 to 12.49	0.5218
	*F*, IL	Normal	Student’s *t* test	−4.41 to 6.75	0.6418
	*F*, PrL	Normal	Student’s *t* test	−9.10 to 10.10	0.9078
	*F*, Cg	Normal	Student’s *t* test	−7.95 to 8.35	0.9562
	*G*, IL	Normal	Student’s *t* test	−12.13 to 6.43	0.4995
	*G*, PrL	Non-normal	Mann–Whitney *U* test	−5.73 to 13.55	0.5476
	*G*, Cg	Normal	Student’s *t* test	−8.17 to 12.16	0.6624
	*I*, IL	Normal	Student’s *t* test	−19.31 to 7.74	0.3456
	*I*, PrL	Normal	Student’s *t* test	−17.93 to 13.13	0.7257
	*I*, Cg	Normal	Student’s *t* test	−20.41 to 13.74	0.6582
	*K*, IL	Non-normal	Mann–Whitney *U* test	−18.57 to 1.24	0.1605
	*K*, PrL	Normal	Student’s *t* test	−15.33 to −1.79	**0.0171**
	*K*, Cg	Normal	Student’s *t* test	−20.59 to −3.42	**0.0098**

**Table 3:** Statistical table for **Figure 3**

Gene	P21								
	Data structure	Type of test	Confidence interval	*p* value	*t* statistic	Degrees of freedom	*U* score	Median and *n* of vehicle	Median and *n* of PNFlx
*aggrecan*	Normal	Student’s *t* test	−0.45 to 0.36	0.8008	0.2585	11	-	-	-
*neurocan*	Normal	Student’s *t* test	−0.72 to 0.13	0.1531	1.535	11	-	-	-
*brevican*	Normal	Student’s *t* test	−0.73 to 0.29	0.3640	0.9469	11	-	-	-
*versican*	Normal	Student’s *t* test	−0.64 to 0.09	0.1301	1.636	11	-	-	-
*hapln1*	Non-normal	Mann–Whitney *U* test	−0.54 to 0.21	0.8357	-	-	19	0.9922 *n* = 6	0.9661 *n* = 7
*hapln2*	Normal	Student’s *t* test	−0.60 to 0.26	0.4059	0.8643	11	-	-	-
*cspg4*	Normal	Student’s *t* test	−0.39 to 0.21	0.5284	0.6510	11	-	-	-
*tenascin c*	Non-normal	Mann–Whitney *U* test	−0.48 to 0.37	0.9452	-	-	20	1.104 *n* = 6	1.065 *n* = 7
*tenascin r*	Non-normal	Mann–Whitney *U* test	−1.04 to 0.09	0.1014	-	-	9	0.7689 *n* = 6	0.6551 *n* = 7
*has1*	Normal	Student’s *t* test	−0.35 to 0.40	0.8841	0.1492	11	-	-	-
*has2*	Non-normal	Mann–Whitney *U* test	−0.30 to 0.20	0.6282	-	-	17	1.021 *n* = 6	1.085 *n* = 7
*has3*	Normal	Student’s *t* test	−0.40 to 0.19	0.4629	0.7606	11	-	**-**	**-**
*chst3*	Non-normal	Mann–Whitney *U* test	−0.06 to 0.80	0.0653	-	-	22	0.8176 *n* = 10	1.359 *n* = 9
*chst7*	Normal	Student’s *t* test	0.05 to 1.30	**0.0352**	2.288	17	-	-	-
*chst11*	Normal	Student’s *t* test	−0.29 to 1.04	0.2480	1.196	17	-	-	-
*chst12*	Normal	Student’s *t* test	0.09 to 1.00	**0.0226**	2.507	17	-	-	-
*chst13*	Non-normal	Mann–Whitney *U* test	−1.11 to 0.35	0.1520	-	-	15	0.9953 *n* = 8	0.2621 *n* = 7
*timp1*	Normal	Student’s *t* test	−0.37 to 0.19	0.5017	0.6946	11	-	-	-
*timp4*	Non-normal	Mann–Whitney *U* test	−0.41 to 0.17	0.9999	-	-	21	0.9842 *n* = 6	1.045 *n* = 7
*mmp2*	Normal	Student’s *t* test	−0.60 to −0.004	**0.0474**	2.231	11	-	-	-
*mmp9*	Normal	Student’s *t* test	−0.38 to 0.03	0.0881	1.871	11	-	-	-
Gene	Adult								
	Data structure	Type of test	Confidence interval	p value	*t* statistic	Degrees of freedom	*U* score	Median and *n* of vehicle	Median and *n* of PNFlx
*aggrecan*	Normal	Student’s *t* test	−0.43 to 0.03	0.0881	1.817	16	-	-	-
*neurocan*	Non-normal	Mann–Whitney *U* test	−0.34 to 0.94	0.3702	-	-	37	0.9189 *n* = 11	1.014 *n* = 9
*brevican*	Normal	Student’s *t* test	−0.17 to 0.65	0.2335	1.238	16	-	-	-
*versican*	Normal	Student’s *t* test	−0.20 to 0.10	0.4973	0.6956	15	-	-	-
*hapln1*	Normal	Student’s *t* test	−0.22 to −0.06	0.2205	1.275	16	-	-	-
*hapln2*	Normal	Student’s *t* test	0.35 to 0.86	0.1137	1.673	16	-	-	-
*cspg4*	Non-normal	Mann–Whitney test	−0.16 to 0.92	0.2799	-	-	35	1.084 *n* = 10	1.118 *n* = 10
*tenascin c*	Normal	Student’s *t* test	−0.32 to −0.20	**0.0054**	3.219	16	-	-	-
*tenascin r*	Normal	Student’s *t* test	−0.93 to 0.42	0.4397	0.8507	16	-	-	-
*has1*	Normal	Student’s *t* test	−0.30 to −0.10	0.1810	1.399	16	-	-	-
*has2*	Normal	Student’s *t* test	−0.30 to −0.09	0.2027	1.328	16	-	-	-
*has3*	Normal	Student’s *t* test	−0.40 to −0.24	**0.0129**	2.797	16	-	**-**	**-**
*chst3*	Non-normal	Mann–Whitney *U* test	−0.56 to 0.51	0.9999	-	-	36	0.8699 *n* = 9	0.8299 *n* = 8
*chst7*	Non-normal	Mann–Whitney *U* test	−0.45 to 0.38	0.8884	-	-	34	0.9374 *n* = 9	0.7781 *n* = 8
*chst11*	Normal	Student’s *t* test	−0.62 to 0.48	0.7858	0.2766	15	-	-	-
*chst12*	Non-normal	Mann–Whitney *U* test	−0.50 to 0.28	0.6058	-	-	30	0.8976 *n* = 9	0.7979 *n* = 8
*chst13*	Non-normal	Mann–Whitney *U* test	−0.85 to 0.42	0.6058	-	-	30	0.8026 *n* = 9	0.8100 *n* = 8
*timp1*	Normal	Student’s *t* test	−0.04 to 0.26	0.6473	0.4649	19	-	-	-
*timp4*	Normal	Student’s *t* test	−0.15 to 0.54	0.7113	0.3760	18	-	-	-
*mmp2*	Non-normal	Mann–Whitney *U* test	−0.69 to 1.04	0.1564	-	-	27	1.029 *n* = 9	0.5079 *n* = 10
*mmp9*	Non-normal	Mann–Whitney *U* test	−0.43 to 0.50	0.9742	-	-	59	0.9411 *n* = 12	0.8429 *n* = 10

## Results

### PNFlx treatment alters PNN numbers in the hippocampus

**Figure 1.**
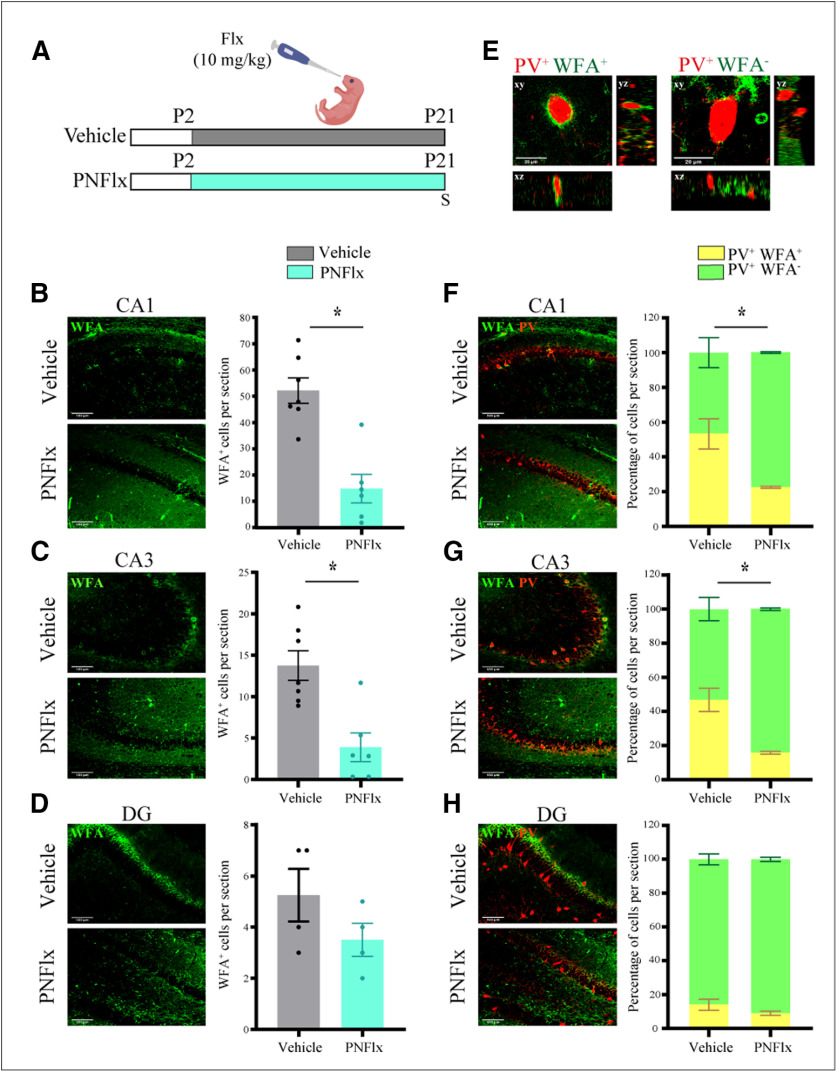
Influence of PNFlx treatment on PNNs in the hippocampus at P21. ***A***, Shown is a schematic depicting the experimental paradigm used to test the effects of PNFlx administration from P2 to P21 on hippocampal PNNs. WFA staining was performed to detect PNNs and to assess the number of PV-positive neurons that were surrounded by PNNs. S denotes the age (P21) at which animals were killed. ***B***, Shown are representative images and quantification of WFA-positive PNNs in the CA1 hippocampal subfields from vehicle-treated and PNFlx-treated animals at P21. PNNs in the CA1 hippocampal subfields of PNFlx animals showed a significant decline compared with vehicle-treated animals. ***C***, Shown are representative images and quantification of WFA-positive PNNs in the CA3 hippocampal subfields from vehicle-treated and PNFlx-treated animals at P21. PNNs in the CA3 hippocampal subfields of PNFlx animals showed a significant decline compared with vehicle-treated animals. ***D***, Shown are representative images and quantification of WFA-positive PNNs in the DG hippocampal subfields from vehicle-treated and PNFlx-treated animals at P21. No significant difference was seen in the number of PNNs in the DG hippocampal subfields of vehicle-treated and PNFlx-treated animals at P21. ***E***, Shown are representative confocal z-stacks of PV-positive neurons which exhibit the presence (left) or absence (right) of co-localization with a WFA-stained PNN. ***F***, Shown are representative double immunofluorescence images of WFA-positive PNNs (green) and PV-positive (red) neurons in the CA1 hippocampal subfields from vehicle-treated and PNFlx-treated animals at P21. Significantly lesser percent of PV-positive cells are surrounded by PNNs in the CA1 hippocampal subfields of PNFlx animals compared with vehicle-treated animals. ***G***, Shown are representative double immunofluorescence images of WFA-positive PNNs (green) and PV-positive (red) neurons in the CA3 hippocampal subfields from vehicle-treated and PNFlx-treated animals at P21. Significantly lesser percent of PV-positive cells are surrounded by PNNs in the CA3 hippocampal subfields of PNFlx animals compared with vehicle-treated animals. ***H***, Shown are representative double immunofluorescence images and quantification of WFA-positive PNNs (green) and PV-positive (red) neurons in the DG hippocampal subfields from vehicle-treated and PNFlx-treated animals at P21. Quantification revealed no significant difference between the DG hippocampal subfields of vehicle-treated and PNFlx-treated animals at P21. Results are expressed as the mean ± SEM; *p <* *0.05 as compared with vehicle-treated control animals, two-tailed unpaired Student’s *t* test or Mann–Whitney *U* test.

**Figure 2.**
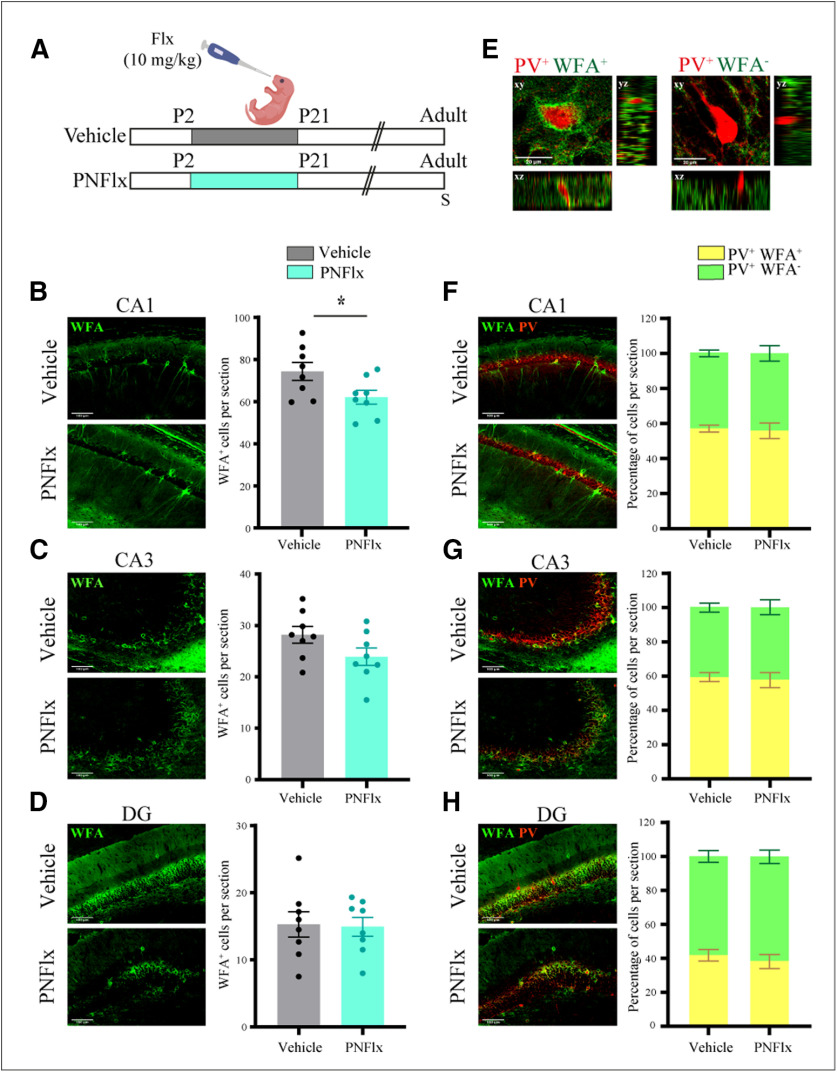
Influence of PNFlx treatment on PNNs in the hippocampus in adulthood. ***A***, Shown is a schematic depicting the experimental paradigm used to test the effects of PNFlx administration from P2 to P21 on hippocampal PNNs. WFA staining was performed to detect PNNs and to assess the number of PV-positive neurons that were surrounded by PNNs. S denotes the age (P80) at which animals were killed. ***B***, Shown are representative images and quantification of WFA-positive PNNs in the CA1 hippocampal subfields from vehicle-treated and PNFlx-treated adult animals. PNNs in the CA1 hippocampal subfields of PNFlx animals showed a significant decline compared with vehicle-treated animals. ***C***, Shown are representative images and quantification of WFA-positive PNNs in the CA3 hippocampal subfields from vehicle-treated and PNFlx-treated adult animals. No significant difference was seen in the number of PNNs in the CA3 hippocampal subfields of vehicle-treated and PNFlx-treated animals in adulthood. ***D***, Shown are representative images and quantification of WFA-positive PNNs in the DG hippocampal subfields from PNFlx-treated and vehicle-treated adult animals. No significant difference was seen in the number of PNNs in the DG hippocampal subfields of vehicle-treated and PNFlx-treated animals in adulthood. ***E***, Shown are representative confocal z-stacks of PV-positive neurons which exhibit the presence (left) or absence (right) of co-localization with a WFA-stained PNN. ***F***, Shown are representative double immunofluorescence images and quantification of WFA-positive PNNs (green) and PV-positive (red) neurons in the CA1 hippocampal subfields from vehicle-treated and PNFlx-treated adult animals. ***G***, Shown are representative double immunofluorescence images and quantification of WFA-positive PNNs (green) and PV-positive (red) neurons in the CA3 hippocampal subfields from vehicle-treated and PNFlx-treated adult animals. ***H***, Shown are representative double immunofluorescence images and quantification of WFA-positive PNNs (green) and PV-positive (red) neurons in the DG hippocampal subfields from vehicle-treated and PNFlx-treated adult animals. Quantification revealed no significant difference between the CA1, CA3, and DG hippocampal subfields of vehicle-treated and PNFlx-treated animals in adulthood. Results are expressed as the mean ± SEM; *p <* *0.05 as compared with vehicle-treated control animals, two-tailed unpaired Student’s *t* test or Mann–Whitney *U* test.

PNNs are ECM-based structures that play a key role in the maturation of neuronal circuits, and are known to exhibit substantial alterations in response to environmental perturbations during critical period windows (Sorg et al., 2016). To study the effects of PNFlx exposure on PNNs in the hippocampal subfields, we orally administered fluoxetine to rat pups from P2 to P21 (Figs. 1*A*, 2*A*). At P21 and in adulthood, we examined the expression of WFA, a plant lectin that binds to the sugar moieties present on the PNNs (Brückner et al., 1996), to visualize PNN numbers. This allowed us to address the short and long-term influence of elevation of serotonin levels during the postnatal temporal window via PNFlx treatment on both the formation and maintenance of PNNs in the hippocampus.

At P21, we observed a robust decline in the number of WFA-stained PNNs in the CA1 (*p *=* *0.0003, *n* = 6–7 per group; Fig. 1*B*) and CA3 (*p *=* *0.0023, *n* = 6–7 per group; Fig. 1*C*) hippocampal subfields in PNFlx animals compared with the vehicle-treated controls. In contrast, we noted no difference in the number of PNNs detected within the DG subfield (Fig. 1*D*). PNNs condense primarily around local GABAergic PV-positive interneurons across numerous cortical and subcortical regions, restricting extensive experience-dependent remodeling within these circuits (Hensch, 2004). We next analyzed whether PNFlx treatment influenced the fraction of hippocampal PV-positive interneurons that were surrounded by PNNs (Fig. 1*E*) at the early time point of P21. We find a significantly smaller fraction of PV-positive neurons surrounded by PNNs in the CA1 (PNN/PV_Vehicle/CA1_ = 53.25%, PNN/PV_PNFlx/CA1_ = 23.25%; *p *=* *0.0286, *n* = 4 per group; Fig. 1*F*) and CA3 (PNN/PV_Vehicle/CA3_ = 46.75%, PNN/PV_PNFlx/CA3_ = 15.75%; *p *=* *0.0286, *n* = 4 per group; Fig. 1*G*) subfields of PNFlx-treated animals than in the vehicle-treated controls. Within the DG subfield, we noted no change in the colocalization of PV-positive neurons with WFA-positive PNNs as a consequence of PNFlx treatment (Fig. 1*H*). Our results indicate that there are marked changes in the total numbers of PNNs, as well as in the colocalization of PV-positive neurons with PNNs, within specific hippocampal subfields immediately after the cessation of PNFlx treatment.

We next sought to address whether the decline in PNN numbers noted in specific hippocampal subfields with PNFlx treatment at P21 persists into adulthood. We find that the CA1 hippocampal subfield of PNFlx animals continued to exhibit a deficit in the total number of PNNs (*p *=* *0.0379, *n* = 8 per group; Fig. 2*B*). In contrast, the number of PNNs in the CA3 hippocampal subfield in the PNFlx cohort did not differ from vehicle-treated controls, indicating that the decline noted in PNN numbers in this subfield at P21 had shown substantial recovery by adulthood (Fig. 2*C*). There was no difference in the number of PNNs in the DG hippocampal subfield in adulthood in the PNFlx cohort (Fig. 2*D*). We did not observe a difference in the proportion of PV-positive neurons that were surrounded by PNNs in either the CA1, CA3, or DG hippocampal subfield (Fig. 2*F–H*). We noted that the WFA-stained PNNs in adulthood (Fig. 2*E*) were better formed and showed a more prominent structure as compared with those at P21 (Fig. 1*E*). This is consistent with age-dependent maturation of the PNN structure that has been reported in many areas of the brain previously (Pizzorusso et al., 2002; Lipachev et al., 2019). Our results suggest that a transient elevation of serotonin in the postnatal temporal window alters the time course of the appearance and maturation of PNNs around PV-positive neurons in a subfield-specific manner in the hippocampus.

### PNFlx treatment alters PNN associated gene expression in the hippocampus

**Figure 3.**
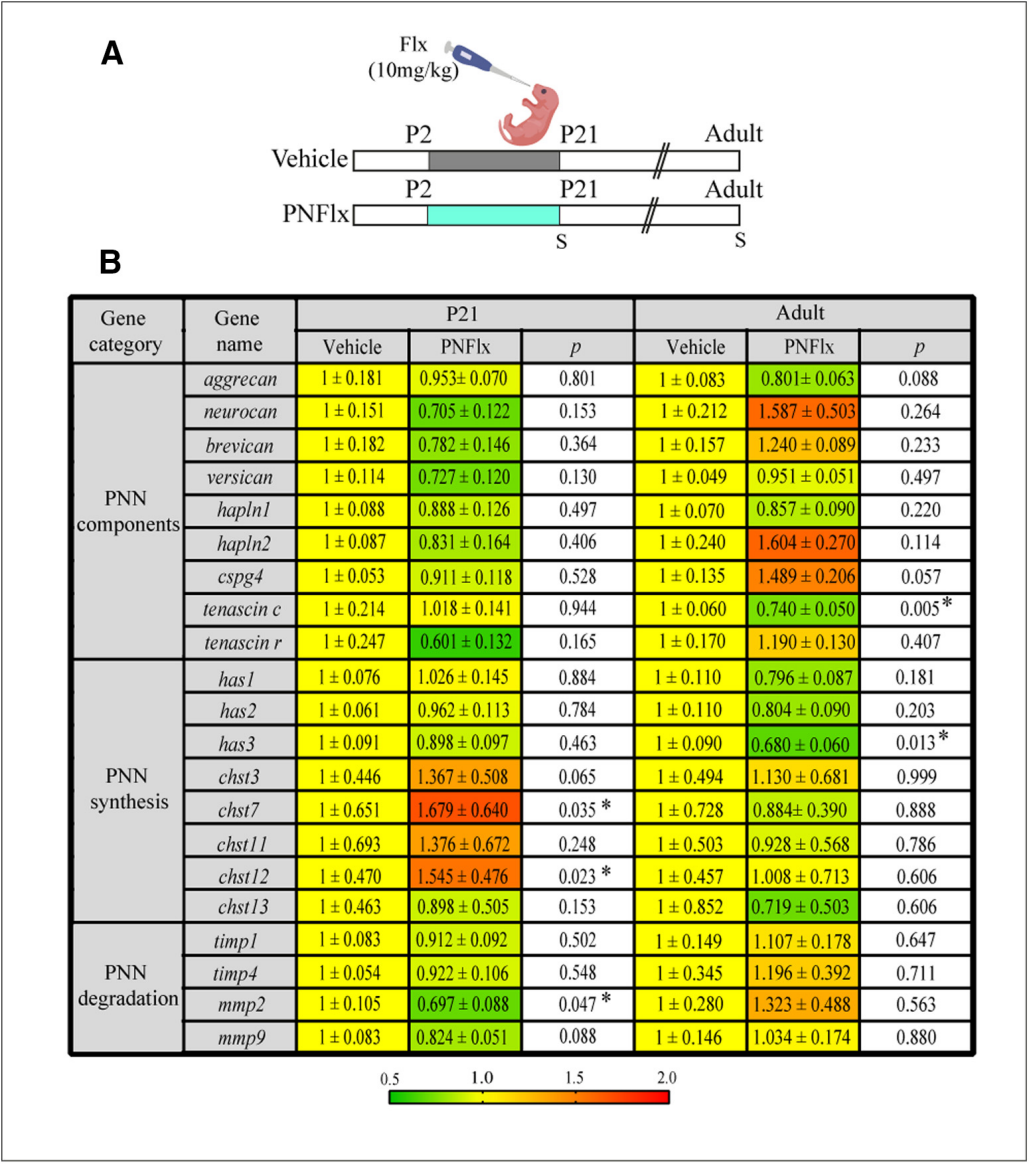
Transcriptional changes of ECM components in adult animals with a history of postnatal administration of fluoxetine. ***A***, Shown is a schematic depicting the experimental paradigm used to test the effects of PNFlx administration from P2 to P21 on expression of PNN associated components in the hippocampus of animals at P21 and adulthood. S denotes the ages (P21 and P100) at which animals were killed. ***B***, Shown are the normalized gene expression levels expressed as fold change relative to their age-matched vehicle-treated animals, as well as *p* values calculated using two-tailed, unpaired Student’s *t* test or Mann–Whitney *U* test (*n* = 6–11 per group). Heat map indicates the extent of gene regulation. Results are expressed as mean ± SEM (*hapln1*, hyaluronan and proteoglycan link protein 1; *hapln2*, hyaluronan and proteoglycan link protein 2; *timp1*, tissue inhibitor of metalloproteinases 1; *timp4*, tissue inhibitor of metalloproteinases 4; *cspg4*, chondroitin sulfate proteoglycan 4; *has1*, hyaluronan synthase 1; *has2*, hyaluronan synthase 2; *has3*, hyaluronan synthase 3; *mmp2*, matrix metalloproteinase 2; *mmp9*, matrix metalloproteinase 9; *chst3*, carbohydrate sulfotransferase 3; *chst7*, carbohydrate sulfotransferase 7; *chst11*, carbohydrate sulfotransferase 11; *chst12*, carbohydrate sulfotransferase 12; *chst13*, carbohydrate sulfotransferase 13).

We next addressed whether the transcription of PNN components or molecular machinery that is known to enzymatically modulate the ECM were specifically altered as a consequence of PNFlx treatment. We performed qPCR analysis on hippocampal lysates from PNFlx-treated and vehicle-treated animals at P21 and in adulthood to examine potential short and long-term changes in the expression of multiple genes associated with ECM composition, synthesis, proteolytic breakdown, sulfation levels and PNN-linked plasticity (Fig. 3*A*,*B*). We examined the expression of genes that encode PNN components (*aggrecan*, *neurocan*, *brevican*, *versican*; hyaluronan and proteoglycan link proteins, *hapln1*, *hapln2*; chondroitin sulfate proteoglycan, *cspg4*, *tenascin c*, and *tenascin r*), enzymatic machinery associated with synthesis of the PNNs (HA synthases, *has1*, *has2*, *has3*, and chondroitin 4-O sulfotransferases, *chst3*, *chst7*, *chst11*, *chst12*, *chst13*), as well as pathways linked to proteolytic degradation of PNNs (tissue inhibitor of metalloproteinases, *timp1*, *timp4*; and matrix metalloproteinases, *mmp2*, *mmp9*; Foscarin et al., 2017; Fawcett et al., 2019). We did not observe any dysregulation in the constituent proteoglycans of PNNs such as *aggrecan*, *brevican*, *neurocan*, *versican*, and *cspg4* in the hippocampus at either age following PNFlx treatment. However at P21, we did observe a downregulation of *mmp2* mRNA (*p *=* *0.0474, *n* = 6–7 per group), a matrix metalloprotease whose role in dendritic remodeling, synaptic plasticity, and axonal sprouting in the hippocampus has been extensively studied (Fujioka et al., 2012). Additionally, we also noted an upregulation in the levels of *chst7* (*p* = 0.0352, *n* = 9–10 per group) and *chst12* transcripts (*p* = 0.0226, *n* = 9–10 per group), which determines the levels of sulfation of the ECM. In adulthood, we observed significant downregulation in the gene expression of *tenascin c*, a glycoprotein, implicated in hippocampal plasticity (Evers et al., 2002; *p *=* *0.005, *n* = 8–10 per group)*. Has3*, an enzyme involved in the synthesis of a critical PNN ECM component, hyaluronan (Fawcett et al., 2019), was also downregulated in the hippocampi of PNFlx animals in adulthood (*p *= 0.0129, *n* = 8–10 per group). Taken together, our data suggest that while PNFlx treatment adversely affects hippocampal PNNs in both a short and long-term manner, there is a limited regulation noted of PNN-associated components at the transcriptional level at these time points.

### Influence of PNFlx treatment on interneuron numbers in the hippocampus

GABAergic interneurons continue to migrate, integrate into circuits, and undergo apoptosis during early postnatal life (Wonders and Anderson, 2006). This temporal window overlaps with the period of PNFlx administration. Interneurons are known to express 5HT_3A_ receptors and the *in vitro* migratory patterns of embryonic interneurons have been reported to be affected by serotonergic signaling (Riccio et al., 2009; Puig and Gulledge, 2011). We thus aimed to investigate the effects of PNFlx treatment on hippocampal interneuron numbers at P21 and in adulthood. We performed immunostaining for four major, non-overlapping subclasses of interneurons, that are immunoreactive for SST, PV, CalR, and Reelin.

**Figure 4.**
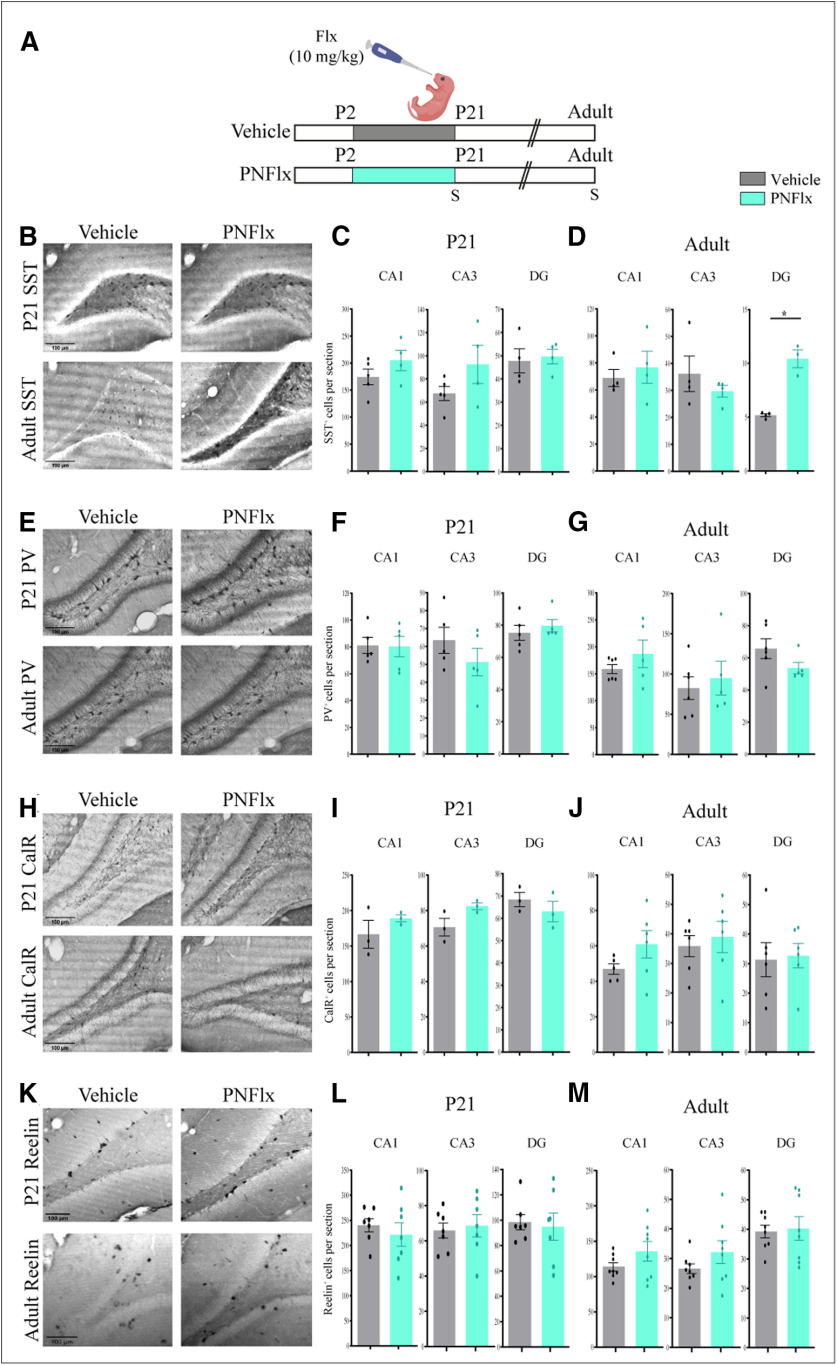
Influence of PNFlx administration on interneuron numbers in the postnatal and adult hippocampus. ***A***, Shown is a schematic depicting the experimental paradigm to test effects of PNFlx administration from P2 to P21 on hippocampal interneurons at P21 and adulthood. S denotes the ages (P21 and P80) at which animals were killed. ***B***, Shown are representative images of SST-positive neurons in the hippocampus of PNFlx-treated and vehicle-treated animals at P21 and in adulthood. ***C***, Shown is quantification of SST-positive neurons in the CA1, CA3, and DG hippocampal subfields in PNFlx-treated and vehicle-treated control animals at P21. Quantification of SST-positive neurons in the CA1, CA3, and DG hippocampal subfields did not show a significant change between PNFlx-treated and vehicle-treated animals at P21. ***D***, Shown is quantification of SST-positive neurons in the CA1, CA3, and DG hippocampal subfields in PNFlx-treated and vehicle-treated animals in adulthood. Quantification of SST-positive neurons showed a significant increase in the DG hippocampal subfield of adult PNFlx-treated animals while no change was observed in the CA1 and CA3 hippocampal subfields. ***E***, Shown are representative images of PV-positive neurons in the hippocampus of PNFlx-treated and vehicle-treated animals at P21 and in adulthood. ***F***, Shown is quantification of PV-positive neurons in the CA1, CA3, and DG hippocampal subfields in PNFlx-treated and vehicle-treated animals at P21. Quantification of PV-positive neurons in the CA1, CA3, and DG hippocampal subfields did not show a significant change between PNFlx-treated and vehicle-treated animals at P21. ***G***, Shown is quantification of PV-positive neurons in the CA1, CA3, and DG hippocampal subfields in PNFlx-treated and vehicle-treated animals in adulthood. Quantification of PV-positive neurons in the CA1, CA3, and DG hippocampal subfields did not show a significant change between PNFlx-treated and vehicle-treated animals in adulthood. ***H***, Shown are representative images of CalR-positive neurons in the hippocampus of PNFlx-treated and vehicle-treated animals at P21 and in adulthood. ***I***, Shown is quantification of CalR-positive neurons in the CA1, CA3, and DG hippocampal subfields in PNFlx-treated and vehicle-treated animals at P21. Quantification of CalR-positive neurons in the CA1, CA3, and DG hippocampal subfields did not show a significant change between PNFlx-treated and vehicle-treated animals at P21. ***J***, Shown is quantification of CalR-positive neurons in the CA1, CA3, and DG hippocampal subfields in PNFlx-treated and vehicle-treated animals in adulthood. Quantification of CalR-positive neurons in the CA1, CA3, and DG hippocampal subfields did not show a significant change between PNFlx-treated and vehicle-treated animals in adulthood. ***K***, Shown are representative images of Reelin-positive neurons in the hippocampus of PNFlx-treated and vehicle-treated animals at P21 and in adulthood. ***L***, Shown is quantification of Reelin-positive neurons in the CA1, CA3, and DG hippocampal subfields in PNFlx-treated and vehicle-treated animals at P21. Quantification of Reelin-positive neurons in the CA1, CA3, and DG hippocampal subfields did not show a significant change between PNFlx-treated and vehicle-treated animals at P21. ***M***, Shown is quantification of Reelin-positive neurons in the CA1, CA3, and DG hippocampal subfields in PNFlx-treated and vehicle-treated animals at adulthood. Quantification of Reelin-positive neurons in the CA1, CA3, and DG hippocampal subfields did not show a significant change between PNFlx-treated and vehicle-treated animals in adulthood. All results are expressed as the mean ± SEM; *p <* *0.05 as compared with vehicle-treated animals, two-tailed unpaired Student’s *t* test or Mann–Whitney *U* test.

At P21, we observed no significant difference in the number of SST, PV, CalR, and Reelin-positive interneurons in the hippocampi of PNFlx-treated animals compared with vehicle-treated controls (Fig. 4*C*,*F*,*I*,*L*). Given we noted a significant decline in the number of PV-positive neurons surrounded by PNNs at P21 in the CA1 hippocampal subfield, this indicates that PNFlx impacts the formation of PNNs but does not influence total PV-positive neuron number. Similarly, in adulthood, the numbers of PV-positive, CalR-positive, and Reelin-positive interneurons present in the CA1, CA3, and DG hippocampal subfields (Fig. 4*D*,*G*,*J*,*M*) was not altered by PNFlx treatment. However, we did observe a small but significant increase in the number of SST-positive neurons in the DG (Fig. 4*D*), but not the CA subfields, of the hippocampus in adult animals with a history of PNFlx treatment (*p *=* *0.0007, *n* = 3–4 per group).

Taken together, our results indicate that PNFlx treatment influences the formation of PNNs in the hippocampus during postnatal development. The changes arise in the absence of any major effect on the total numbers of hippocampal SST, PV, CalR, and Reelin-positive interneurons, with the exception of a small but significant increase in SST neuron number in the DG subfield of PNFlx animals in adulthood.

### Adult animals with a history of PNFlx treatment exhibit a decline in hippocampal GABA-Aα2 expression and enhanced c-Fos expression

**Figure 5.**
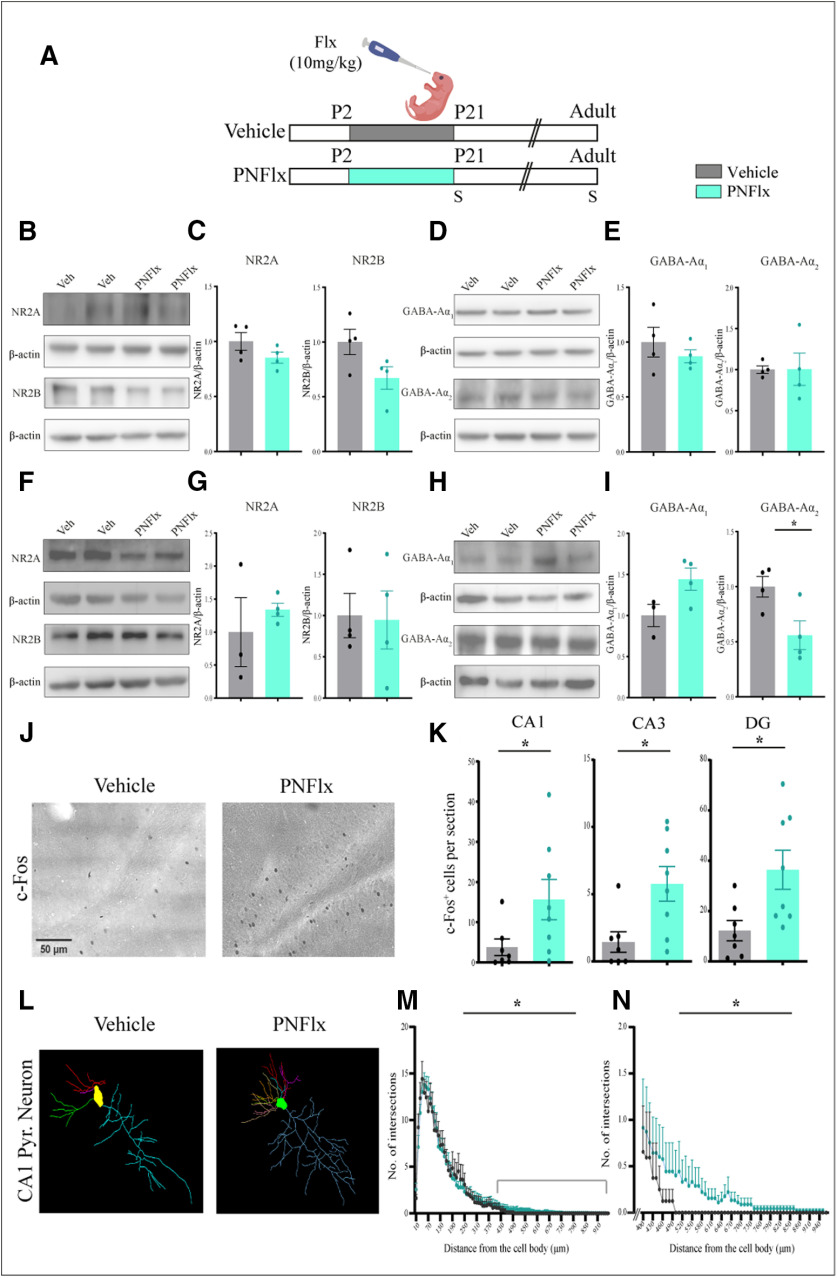
Influence of PNFlx treatment on NMDA and GABA receptor subunit expression, c-Fos-positive cell numbers and CA1 pyramidal neuron morphology in the hippocampus. ***A***, Shown is a schematic depicting the experimental paradigm to test the effects of PNFlx administration from P2 to P21 on hippocampal NMDA and GABA receptor subunit expression, c-Fos expression, and architecture of CA1 pyramidal neurons in adult animals. S denotes the age (P21 and P80–P120) at which animals were killed. ***B***, Shown are representative Western blottings of NR2A and NR2B with β-Actin as the loading control from the hippocampus of vehicle-treated and PNFlx-treated animals at P21. ***C***, Shown are densitometric quantifications of normalized protein levels of NR2A and NR2B. Normalized levels of NR2A and NR2B showed no difference between vehicle-treated and PNFlx-treated animals at P21. ***D***, Shown are representative Western blottings of GABA-Aα1 and GABA-Aα2 with β-Actin as the loading control from the hippocampus of vehicle-treated and PNFlx-treated at P21. ***E***, Shown are densitometric quantification of normalized protein levels of GABA-Aα1 and GABA-Aα2. Normalized levels of GABA-Aα1 and GABA-Aα2 showed no difference between vehicle-treated and PNFlx-treated animals at P21. ***F***, Shown are representative Western blottings of NR2A and NR2B with β-Actin as the loading control from the hippocampus of vehicle-treated and PNFlx-treated adult animals. ***G***, Shown are densitometric quantifications of normalized protein levels of NR2A and NR2B. Normalized levels of NR2A and NR2B showed no difference between vehicle-treated and PNFlx-treated animals in adulthood. ***H***, Shown are representative Western blottings of GABA-Aα1 and GABA-Aα2 with β-Actin as the loading control from the hippocampus of vehicle-treated and PNFlx-treated adult animals. ***I***, Shown are densitometric quantification of normalized protein levels of GABA-Aα1 and GABA-Aα2. Normalized levels of GABA-Aα2 showed a significant decrease in the hippocampus of PNFlx-treated adult animals compared with vehicle-treated adult animals while no significant difference was seen in GABA-Aα1 between vehicle-treated and PNFlx-treated animals in adulthood. ***J***, Shown are representative images of c-Fos expressing cells in the hippocampus from vehicle-treated and PNFlx-treated animals in adulthood. ***K***, Quantifications of c-Fos expressing cells showed a significant increase within the hippocampal subfields of CA1, CA3, and DG in adult PNFlx-treated animals. ***L***, Shown are representative traces of Golgi–Cox-stained CA1 hippocampal pyramidal neurons of vehicle-treated and PNFlx-treated adult animals. ***M***, Shown is quantification of the number of intersections per micrometer of distance from the soma across the entire apical dendritic arbor in Golgi–Cox-stained hippocampal CA1 pyramidal neurons of PNFlx-treated and vehicle-treated adult animals. ***N***, Shown is quantification of the number of intersections per μm distance from the soma across the distal end (400–940 μm) of the apical arbors of Golgi–Cox-stained hippocampal CA1 pyramidal neurons of PNFlx-treated and vehicle-treated adult animals. Results for Western blottings, c-Fos immunohistochemistry, total number of intersections of Golgi–Cox-stained CA1 pyramidal neurons are expressed as the mean ± SEM; *p <* *0.05 as compared with vehicle-treated rats using two-tailed, unpaired Student’s *t* test. Results for number of intersections and number of intersections per um distance from the soma of Golgi–Cox-stained CA1 pyramidal neurons are expressed as the mean ± SEM and PNFlx animals have been compared with vehicle-treated animals using non-parametric repeated measures Friedman’s test.

Given we noted changes in PNN numbers following PNFlx treatment accompanied by ECM-associated gene expression changes within the hippocampus, we sought to address whether receptor-subunit switches of the NMDA and GABA receptors, associated with maturation of hippocampal circuitry (Davis et al., 2000; Gambrill and Barria, 2011), are disrupted as a result of PNFlx treatment. PNNs are known to prevent the lateral movement and exchange of transmembrane receptor molecules (Sorg et al., 2016). We examined two classic maturation associated receptor-subunit switches, namely the levels of hippocampal expression of NR2A and NR2B, and GABA-Aα1 and GABA-Aα2, in vehicle-treated and PNFlx-treated animals at P21 (Fig. 5*A–E*) and in adulthood (Fig. 5*A*,*F–I*). Western blot analysis revealed that NR2A, NR2B, GABA-Aɑ1, and GABA-Aɑ2 protein levels in the hippocampus are unaltered at P21 (Fig. 5*B–E*). In adulthood, the protein levels of NR2A, NR2B, and GABA-Aɑ1 remain unchanged (Fig. 5*F–I*), whereas GABA-Aɑ2 was significantly reduced within the hippocampi of adult animals with a history of PNFlx treatment (*p *=* *0.033, *n* = 4 per group; Fig. 5*H*,*I*).

We then addressed whether the altered PNN formation, perturbed gene expression of ECM-associated molecules and dysregulation of GABA-Aα2 expression in the hippocampi of PNFlx animals was also associated with any change in baseline neuronal activity levels by assessing the influence of PNFlx on expression of the neuronal activity marker, c-Fos (Bullitt, 1990). Analysis of c-Fos expression in the hippocampus revealed that adult animals with a history of PNFlx treatment exhibited significantly higher numbers of c-Fos-positive cells in the hippocampal CA1 (*p *=* *0.0175, *n* = 7–8 per group), CA3 (*p *=* *0.0105, *n* = 7–8 per group), and DG (*p *=* *0.017, *n* = 7–8 per group) subfields (Fig. 5*J*,*K*). These observations indicate that baseline neuronal activity within the hippocampus is increased in adult animals with a history of PNFlx treatment.

Previous evidence indicates that plasticity of PNNs encapsulating PV-positive neurons can directly impact both excitation and cytoarchitecture of output pyramidal neurons (Orlando et al., 2012). Given that neuronal architecture is known to be strongly correlated with changes in neuronal activity, and is also dependent on the surrounding ECM (Levy et al., 2014), we addressed whether PNFlx treatment led to persistent changes in dendritic morphology of the principal CA1 pyramidal neurons. To address this, we used Golgi–Cox staining to trace the processes of CA1 pyramidal neurons in vehicle-treated and PNFlx-treated adult animals. Sholl analysis revealed a small, but significant, increase in dendritic arbors at the distal-most ends of the apical branches of CA1 pyramidal neurons as a consequence of PNFlx treatment (*p *<* *0.0001, *n* = 8–9 per group; Fig. 5*L–N*). We have not examined the influence of PNFlx treatment on spine density, or differences in morphology that may be noted at earlier time points.

Collectively, our results indicate that PNFlx treatment evokes a delayed development of PNNs in the hippocampus during the postnatal window and results in persistent changes in neuronal activity, GABA-A receptor subunit composition and CA1 pyramidal neuron dendritic complexity that are noted in adulthood long after the cessation of PNFlx treatment.

### Influence of PNFlx treatment on interneuron, PNN, and c-Fos-positive cell numbers within the mPFC

**Figure 6.**
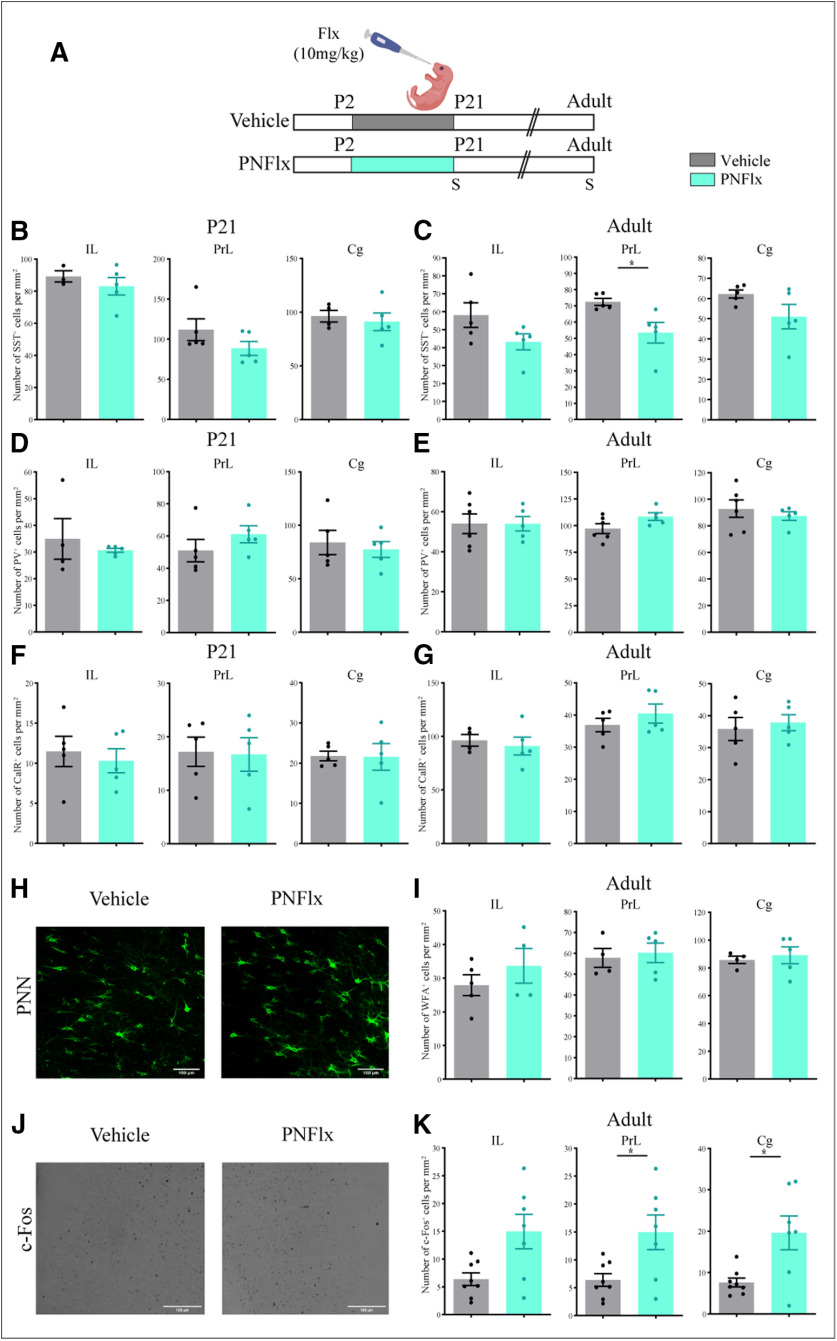
Influence of PNFlx treatment on interneuron numbers, PNNs, and c-Fos-positive cells in the mPFC. ***A***, Shown is a schematic depicting the experimental paradigm to test the effects of PNFlx administration from P2 to P21 on interneuron numbers, PNNs, and the number of c-Fos-positive cells. S denotes the age (P21 and P80) at which animals were killed. ***B***, Shown is the quantification of SST-positive neurons in the IL, PrL, and Cg subdivisions of the mPFC in PNFlx-treated and vehicle-treated control animals at P21. ***C***, Shown is the quantification of SST-positive neurons in the IL, PrL, and Cg subdivisions of the mPFC in PNFlx-treated and vehicle-treated control animals at P80. Quantification of SST-positive neurons showed a significant decrease in the PrL subfield of the mPFC in adult PNFlx-treated animals while no change was observed in IL and Cg subdivisions. ***D***, Shown is the quantification of PV-positive neurons in the IL, PrL, and Cg subdivisions of the mPFC in PNFlx-treated and vehicle-treated control animals at P21. ***E***, Shown is the quantification of PV-positive neurons in the IL, PrL, and Cg subdivisions of the mPFC in PNFlx-treated and vehicle-treated control animals at P80. ***F***, Shown is the quantification of CalR-positive neurons in the IL, PrL, and Cg subdivisions of the mPFC in PNFlx-treated and vehicle-treated control animals at P21. ***G***, Shown is the quantification of CalR-positive neurons in the IL, PrL, and Cg subdivisions of the mPFC in PNFlx-treated and vehicle-treated control animals at P80. ***H***, Shown are representative images of WFA-positive PNNs in the Cg subdivision of the mPFC of PNFlx-treated and vehicle-treated animals in adulthood. ***I***, Shown is the quantification of WFA-positive PNNs in the IL, PrL, and Cg subdivisions of the mPFC in PNFlx-treated and vehicle-treated control animals at P80. ***J***, Shown are representative images of c-Fos-positive cells in the PrL subdivision of the mPFC of PNFlx-treated and vehicle-treated animals in adulthood. ***K***, Quantifications of c-Fos-positive cells indicate a significant increase within the PrL, and Cg subdivisions, with no change noted in the IL, subdivision of the mPFC in adult PNFlx-treated animals. All results are expressed as the mean ± SEM; *p <* *0.05 as compared with vehicle-treated animals, two-tailed unpaired Student’s *t* test or Mann–Whitney *U* test.

Given that PNFlx treatment evokes subtle changes in SST-positive interneuron numbers, alters the number of PNNs and is associated with perturbed c-Fos expression in the hippocampus, we next sought to investigate whether similar changes occurred within the mPFC, a key limbic region strongly implicated in anxio-depressive behaviors (Covington et al., 2010; Adhikari et al., 2011; Pati et al., 2018). The mPFC transiently expresses the serotonin transporter in the postnatal window (Lebrand et al., 1998) and has been shown to undergo molecular and architectural changes in response to postnatal administration of SSRIs (Rebello et al., 2014). We first assessed the effects of PNFlx treatment on numbers of distinct interneuronal populations, namely SST, PV, and CalR-positive neurons, within the mPFC at P21 and in adulthood. We noted no difference in the number of SST, PV, and CalR-positive interneurons, in the IL, PrL, and Cg subdivisions of the mPFC at P21 (Fig. 6*B*,*D*,*F*). In adulthood, the number of PV and CalR-positive interneurons were unaltered in the IL, PrL, and Cg subdivisions of the mPFC (Fig. 6*E*,*G*). We did note a small, but significant, decline in the number of SST-positive interneurons in the PrL subdivision of the mPFC in adulthood (*p *=* *0.0216, *n* = 5 per group; Fig. 6*C*).

We next sought to investigate the influence of PNFlx treatment on the expression of PNNs in the mPFC. In this regard, we attempted to assess the influence of PNFlx treatment at P21; however, at this developmental stage, it was difficult to visualize and distinguish PNNs in the mPFC, and hence we have restricted our analysis to the adult time-window. We observed no change in the number of WFA-positive PNNs within the IL, PrL, and Cg (Fig. 6*H*) subdivisions of the mPFC between PNFlx-treated and vehicle-treated animals in adulthood (Fig. 6*I*). We next investigated the influence of PNFlx treatment on baseline neuronal activity via assessing the expression of c-Fos-positive cell numbers within the mPFC subdivisions (Fig. 6*J*). Adult animals with a history of PNFlx treatment exhibited a significant increase in the number of c-Fos-positive cells in the PrL (*p *=* *0.017, *n* = 7–8 per group; Fig. 6*K*), and Cg (*p *=* *0.009, *n* = 7–8 per group; Fig. 6*K*), but not the IL, subdivisions of the mPFC. These results indicate that adult animals with a history of PNFlx treatment have enhanced c-Fos-positive cell numbers in the PrL and Cg mPFC subdivisions suggestive of enhanced neuronal activity in the mPFC.

In summary, we note subtle changes in the number of SST-positive interneuron numbers in both the mPFC and hippocampus, albeit in opposing directions. We also observed that PNFlx treatment evokes perturbed neuronal activity, as reflected by altered c-Fos expression in both the mPFC and the hippocampus in adulthood. These observations motivate future experiments to address whether changes in local microcircuitry within multiple cortical brain regions are persistent long after the cessation of PNFlx treatment.

## Discussion

**Figure 7.**
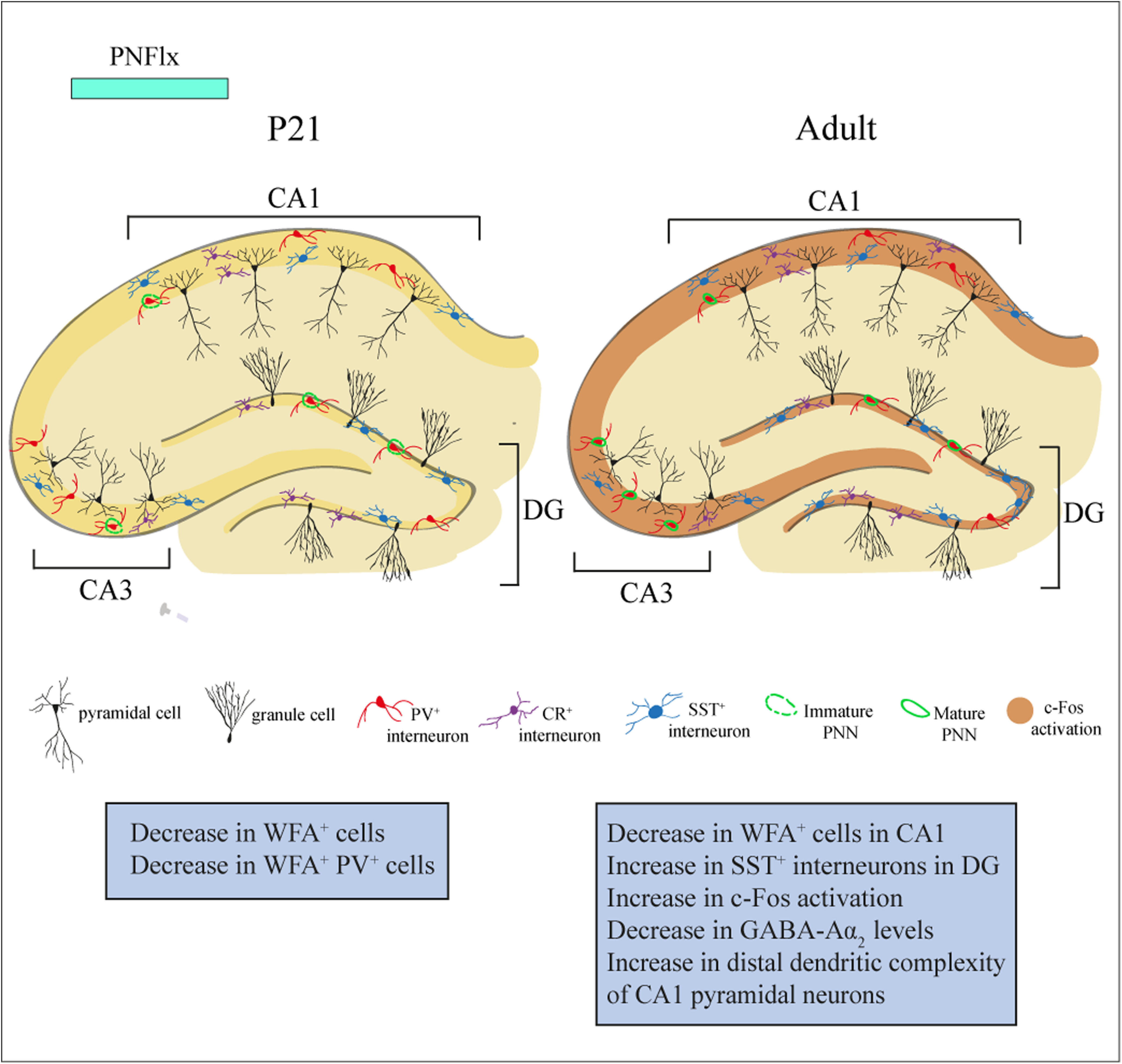
PNFlx treatment causes immediate and long-lasting cellular and molecular changes in the rat hippocampus. PNFlx treatment alters the developmental trajectory of PNN formation within the hippocampus. PNFlx treatment evoked a decline in the number of WFA-positive PNNs, and PV-positive cells ensheathed by WFA-positive PNNs, in the CA1 and CA3 hippocampal subfields at P21. A history of PNFlx treatment was associated with a decline in the number of WFA-positive PNNs in the CA1 hippocampal subfield, and a small increase in the number of SST-positive neurons in the DG hippocampal subfield in adulthood. Adult animals with a history of PNFlx also exhibited an increase in the number of c-Fos-positive cells in all hippocampal subfields, a decline in GABA-Aα2 receptor subunit expression within the hippocampus, and enhanced dendritic complexity noted in the distal dendrites of the apical branches of CA1 pyramidal neurons.

Here, we have performed a detailed characterization of the short and long-term consequences of PNFlx exposure on PNNs, hippocampal interneuron number and regulation of ECM-related gene expression. We find a significant reduction in PNN numbers within the CA1 and CA3 hippocampal subfields, immediately following the cessation of PNFlx treatment at P21, and this decline persists within the CA1 subfield into adulthood. We did not observe any change in the numbers of specific interneuron classes, characterized by the expression of PV, SST, CalR, and Reelin, in the hippocampus at P21. However, we did note a small, but significant, increase in SST-positive interneurons in the DG hippocampal subfield in adulthood in the PNFlx cohort. Our results suggest that PNFlx can influence PNN development in the hippocampus, which is known to exhibit a protracted window of development extending well into postnatal life and adolescence (Bayer, 1980; Rice and Barone, 2000). Given that the temporal window of PNFlx overlaps with substantial neuronal plasticity in terms of interneuron migration, apoptosis, synaptic pruning and synaptogenesis, as well as the formation of PNNs, our results provide an insight into the developmental consequences of enhanced serotonin levels on the maturing hippocampal neurocircuit (Gingrich et al., 2017). Further, we show that PNFlx treatment leads to a reduction in hippocampal GABA-Aɑ2 receptor subunit protein levels, and an increase in c-Fos expression across all hippocampal subfields in adulthood, suggesting persistent dysregulation of neuronal activity in the hippocampus as a consequence of the early life fluoxetine exposure. This is accompanied by increased dendritic complexity in the distal regions of the apical branches of CA1 pyramidal neurons in adult animals with a history of PNFlx. Collectively, our findings reveal both early-onset and persistent changes in PNN formation, interneuron number, neuronal activity, CA1 pyramidal neuron cytoarchitecture (summarized in Fig. 7) and altered transcriptional regulation of ECM and PNN-associated genes in the hippocampus following PNFlx treatment (Fig. 3*B*).

Serotonin modulates the development and maturation of neurons during early life (Gaspar et al., 2003). Perturbations in serotonin levels or signaling during this period can lead to developmental, cytoarchitectural as well as behavioral changes that last well into adulthood (Ansorge et al., 2004; Brummelte et al., 2017; Shah et al., 2018). Early-life SSRI administration has been shown to disrupt PNN formation in multiple limbic circuits (Umemori et al., 2015). A previous study showed that administration of fluoxetine in drinking water to pregnant mouse dams, commencing gestational day 7 and extending until P7, which would presumably result in fluoxetine exposure to nursing pups, showed a significant reduction in PNN numbers in the CA1 hippocampal subfield at P17, and exhibited recovery in cohorts of pups examined at P24 (Umemori et al., 2015). While the present study differs substantially in the mode of fluoxetine administration, treatment timeline, species studied and ages examined, it is note-worthy that we also find a robust decline in PNN numbers at the early time point of P21 following PNFlx treatment. At the P21 time point following PNFlx, we have examined PNN numbers, as well as the number of PV-positive interneurons decorated by PNNs, in both male and female pups. However, our studies in adulthood are restricted to males with a history of PNFlx administration, and this is one of the caveats of our study which does not allow us to comment on whether there are any sexually dimorphic effects on PNNs in adult animals with a history of PNFlx. Studies have also shown that chronic administration of SSRIs in adulthood evokes a juvenile-like plasticity in the visual and PFC associated with a dissolution of PNNs (Maya Vetencourt et al., 2008; Ohira et al., 2013; Guirado et al., 2014). PNN formation, primarily around PV-positive interneurons, is thought to be a critical stage that defines the establishment of the maturity, and emerges at distinct developmental epochs in a circuit-specific manner (Köppe et al., 1997; Lensjø et al., 2017). Our findings indicate that the delayed appearance of PNNs following PNFlx treatment results in significantly fewer numbers of PV-positive interneurons ensheathed by PNNs in the CA1 and CA3 hippocampal subfields at P21. This decline is transient, and by adulthood the number of PV-positive interneurons decorated with PNNs is indistinguishable between vehicle-treated and PNFlx-treated animals. However, our results do not allow us to resolve whether the constituent components of the PNN at these timepoints are perturbed. PNNs play an important role in stabilization of synaptic contacts, structural and functional plasticity, and the establishment of E/I balance in neural circuits (Hensch, 2005; Fawcett et al., 2019). Our results clearly indicate that the establishment of PNNs in specific hippocampal subfields is transiently disrupted during postnatal development as a consequence of PNFlx treatment and that this disruption of PNNs persists into adulthood in a hippocampal subfield-specific manner.

Gene expression analysis revealed that the hippocampal expression of several PNN and ECM-associated genes such as *aggrecan*, *versican*, *brevican*, *neurocan*, *cspg4*, and *hapln1-2* do not exhibit any change in expression at either P21 or adulthood following PNFlx treatment. We did however, observe a decrease in *mmp2*, and an increased expression of *chst7* and *chst12* transcript levels immediately postcessation of the PNFlx treatment at P21, and also noted a long-term decline in *tenascin c* and *has3* mRNA within the hippocampus in adulthood in animals with a history of PNFlx administration. While we cannot draw direct causal links between the transcriptional dysregulation of individual genes to the complex cellular and behavioral phenotypes noted with PNFlx, it is interesting to note that mice constitutively deficient in tenascin C display compromised LTP in CA1 pyramidal neurons (Evers et al., 2002), and mutant mice that were combinatorial knock-outs of *tenascin c*, *tenascin r*, *brevican*, and *neurocan* were shown to have decreased PNN area and intensity in the CA2 hippocampal subfield (Gottschling et al., 2019). Analyses of interneuron numbers revealed that while the numbers of PV, CalR, and Reelin-positive neurons in the hippocampus do not change as a consequence of PNFlx treatment, we do find a significant increase in the number of SST-positive neurons in the DG of adult animals with a history of PNFlx. Multiple studies indicate that perturbed SST-signaling, and a loss of SST-positive neurons are both linked to elevated depressive-like/anxiety-like behaviors, and vulnerability of SST neurons to damage has also been suggested in the context of major depressive disorder (Carmichael and Lockhart, 2012; Lin and Sibille, 2015; Douillard-Guilloux et al., 2017; Fee et al., 2017). Hippocampal SST neurons are also linked to the control of HPA axis activity, with differential effects observed on emotionality based on the class of SST receptors involved (Scheich et al., 2016; Prévôt et al., 2017; Yamamoto et al., 2018). Given the complexity of information processing in hippocampal microcircuitry, it is unclear at present what the consequence of a small increase in SST neuron number in the DG would be on hippocampal function. PNFlx treatment does not appear to result in major disruption of interneuron numbers during postnatal life or in adulthood, however we cannot preclude effects on the cytoarchitecture and function of specific interneuron classes. The decline in PNNs, in particular those that encapsulate PV-positive interneurons, following PNFlx treatment raises the possibility of a disruption of the intrinsic properties of PV-positive interneurons, and hence an impact on the ability of PV-positive interneurons to dynamically gate and adapt their responses to altered neuronal activity (Favuzzi et al., 2017).

Disruption of PNN formation and ECM abnormalities have been linked to the pathophysiology of several psychiatric disorders, including schizophrenia and mood disorders and neurodevelopmental disorders, such as autism spectrum disorder and epilepsy (Pantazopoulos and Berretta, 2016; Wen et al., 2018). Preclinical models of early life trauma that are based on the disruption of dam-pup interactions, such as MS and limited nesting bedding (LNB), perturbed inflammatory milieu evoked via maternal immune activation (MIA) and exposure to juvenile stress have all been reported to evoke a disruption of PNN formation. MS is reported to result in a reduced density of PNNs in the PrL cortex at P20 (Gildawie et al., 2020), and male pups raised by dams in the LNB paradigm exhibit a sexually dimorphic increase in PNN density around PV-positive interneurons within the right basolateral amygdala (Guadagno et al., 2020). Pups born to dams subjected to MIA showed a decline in the density of PNNs in the basolateral amygdala at P35, and in the PrL cortex in adulthood (Paylor et al., 2016). Exposure to juvenile stress is linked to a decrease in the intensity of PNN staining in the CA1 hippocampal subfield, the dorsal anterior Cg cortex, the IL cortex and the motor cortex, immediately following the cessation of the stress (Ueno et al., 2018). Interestingly, MS, LNB, MIA and juvenile stress are all reported to evoke a perturbation of serotonergic signaling (Benekareddy et al., 2010; Luo et al., 2015; Goeden et al., 2016; Jablonski et al., 2017). These results raise the intriguing possibility that both a perturbation of serotonergic signaling, as well as an altered trajectory of PNN development in key limbic circuits, may be associated with the development of anxiogenic and depressive-like behavioral phenotypes noted in adulthood in these models of early adversity. Our studies suggest that PNFlx treatment, which evokes persistent increases in anxiety-like and despair-like behavior across the life-span, also targets the formation of PNNs in the hippocampus, a brain region implicated in the regulation of mood-related behavior. A disruption of molecular and cellular regulators of plasticity during postnatal maturation has been suggested to be a common signature associated with animal models of depression, based on early adversity exposure, as well as genetic and pharmacological models of neurodevelopmental disorders (Fagiolini and Leblanc, 2011; Callaghan et al., 2013; Do et al., 2015; Spijker et al., 2020).

PNN formation and maturation marks the end of the developmental plasticity window in most circuits (Hensch, 2005). A shift in the receptor subunit composition of both NMDA and GABA_A_ receptor subunits is a hallmark signature associated with neurodevelopment and with the establishment of E/I balance (Davis et al., 2000; Gambrill and Barria, 2011). We find that while the ratio of NR2A/NR2B receptor subunits is not altered at P21 or in adult animals with a PNFlx history, we note a robust decline in GABA-Aα2 receptor subunit in the hippocampi of PNFlx-treated animals in adulthood. The natural developmental progression indicates a switch from GABA-Aα2 to GABA-Aα1 receptor subunits in adults (Fritschy et al., 1994), and a change in the ratio of specific GABA-A receptor subunits following PNFlx treatment could have an important implication for neuronal activity within the hippocampal circuitry. Indeed, this is reflected in the c-Fos expression analysis which indicates an increase in expression of the neuronal activity marker in all hippocampal subfields of PNFlx animals in adulthood. Interestingly, we also noted an increase in c-Fos expression within the PrL and Cg subdivisions of the mPFC. Perturbed baseline expression of an immediate early gene marker noted in adulthood in animals with a history of PNFlx administration points to potential disruption of neuronal activity. It will be of interest to also address whether neuronal activity is already perturbed at P21 within the hippocampus and mPFC. Currently, it is difficult to parcellate out whether such a change in neuronal activity in specific limbic brain regions of PNFlx animals is a consequence of mood-related behavioral changes, or whether such altered neuronal activity causally drives the behavioral changes noted with PNFlx. A limitation of our study is that the identity of neurons that are c-Fos-positive is unclear, and hence it remains unknown at present whether animals with a history of PNFlx exhibit enhanced neuronal firing in excitatory or inhibitory neurons within the hippocampus and the mPFC. Our findings motivate further studies to directly test the consequences of PNFlx evoked altered PNN development on the neuronal activity and intrinsic properties of PV-positive interneurons, and on the emergence of E/I balance within the hippocampus and mPFC.

Serotonin is known to modulate neuronal morphology, and dendritic spine shape/density (Daubert and Condron, 2010; Bijata et al., 2017; Wirth et al., 2017). PNFlx treatment from P2 to P11 results in decreased dendritic arbor complexity in pyramidal neurons of the IL cortex (Rebello et al., 2014). In contrast, a developmental knock-out of the serotonin transporter is associated with increased dendritic complexity in IL pyramidal neurons, and an increase in spine density in pyramidal neurons of the basolateral amygdala (Wellman et al., 2007). We noted a subtle increase in dendritic complexity, restricted to the distal-most regions of the apical dendrites of CA1 pyramidal neurons in PNFlx-treated animals in adulthood. Hippocampal gene expression changes associated with PNFlx indicate dysregulation of pathways that modulate neuronal cytoarchitecture, namely mTOR signaling, and perturbed gene expression of the hyperpolarization-activated cyclic nucleotide-gated 1 channel (*Hcn1*), which is known to exhibit distal dendrite enrichment (Sarkar et al., 2014b). While we have not extensively examined the influence of PNFlx on spine number, shape and density in hippocampal pyramidal neurons, our observations motivate a detailed characterization of the impact of early life serotonin elevation on the cytoarchitecture of distinct neuronal classes within the hippocampus.

Collectively, our findings indicate that early life elevation of serotonin via treatment with the SSRI fluoxetine alters the developmental trajectory of PNNs within the hippocampus, suggestive of a disruption of critical period plasticity. This evidence supports an emerging body of literature that implicates disruption of PNNs and ECM dysregulation as putative mechanisms that contribute to the circuit dysfunction associated with psychiatric and neurodevelopmental disorders.

## Synthesis

Reviewing Editor: Carmen Sandi, Swiss Federal Institute of Technology Decisions are customarily a result of the Reviewing Editor and the peer reviewers coming together and discussing their recommendations until a consensus is reached. When revisions are invited, a fact-based synthesis statement explaining their decision and outlining what is needed to prepare a revision will be listed below. The following reviewer(s) agreed to reveal their identity: Eero Castren.

This study on the long-term effects of perinatal fluoxetine in the hippocampus and constitutes an interesting addition to the known effects of this drug on brain wiring during critical developmental periods, with some advances.

Specifically, it focuses on perineuronal nets (PNN) during early life and in the adulthood. The authors found that postnatal (P2-21) fluoxetine treatment decreases the numbers of PNN and the numbers of PV encased by PNNs in hippocampal CA1 and CA3 regions but not the dentate gyrus (DG) at P24. In the adulthood, these effects have essentially been recovered, only in the CA1 region the number is slightly reduced. However, the percentage of PV interneurons surrounded by PNNs does not differ in any of the hippocampal sub regions following fluoxetine treatment in adults. The treatment had essentially no effects on the numbers of interneurons, also the expression of a number of ECM constituents was essentially unaltered. The authors further report an increase in SST interneuron number in the DG during adulthood. Next the authors report decreased expression of the GABA receptor subunit GABA-Aα2 and increased expression of cFos, as well as increased dendritic arbors at the distal-most ends of the apical branches of pyramidal neurons in the CA1.

The developmental effects of perinatal fluoxetine administration have been documented by a number of behavioral and neuroanatomical studies, however consequences on hippocampal development have not been fully explored. The present study fills partly this gap by showing diverse effects: reduction of PNN on PV neurons, changes in GABA-A receptor protein, increase in basal c-fos level and increased dendritic branching. Overall these observations are suggestive of long-term altered excitatory/inhibitory balance in hippocampal circuits.

The paper is very clearly written and presented, and generally well presented. The data are clearly presented and analyzed, and the discussion is fair and correctly places this study in the growing data on this topic.

However, two reviewers and the review editor consider that a few aspects require further attention before publication. Specifically, as indicated below, statistics for figure 3 are incorrect. Concerning the other criticisms listed below, mainly to require more detailed description of the methodology (cell counting) and likely some additional data on c-fos -characterization of cell type and provide info on other brain region. This should not require to do another batch of experiments, but additional investigations on sections the authors have probably kept.

Specifically, see below the list of issues raised by the reviewers:

1. More information is needed on the c-fos experiments; As PNFLX rats may have different behaviours previous to perfusion, the change in c-fos could be a secondary effect. In this regard it would be important to have data on c-fos in other brain regions. This point needs to be adressed.

2. Illustrations: the micrographs in Figure 1: pNN are very hard to see in panels B, C, and D. Contrast could be improved or higher magnifications shown. Figure 1 D: the number of samples used in the analysis of PNN expression in the DG equals 4, while the number of samples used for CA1 and CA3 are n = 6-7. Why are the numbers different? The analysis should be done on the same slices.

3. Schemes of treatment protocol shown in Figure 1 and 2, do not need to be repeated in subsequent figures as there is no change (optional).

4. Figure 2 F, representative figure: vehicle seems to have no co-localization with PV, although the figure shows there is no difference between co-localization in vehicle and fluoxetine-treated samples

5. Figure 3: First, the author should better explain how they selected the genes to be analysed. Why were the enzymes involved in PNN production not checked.

6. The experiment shown in Fig 3 compares 2 factors (age and fluoxetine treatment), so a Two-way ANOVA analysis followed by a post-hoc would be more appropriate than multiple T tests to understand if fluoxetine effect depends on the mice´s age. Moreover, it would then be possible to see how the expression of different extracellular matrix components changes along development. Same suggestion could be applied to other experiments comparing two factors (age and flx), and two-way PERMANOVA (freely available in Past3 software) could be used if a non-parametric test is required.

7. It is noteworthy that differences in the range of 40-60% are not nearly close to statistical significance (e.g. tenascin r, hapln2, neurocan). Could those be false negative due to small n? Please specify also the df and F values (for this and other experiments), so the reader can have a better understanding of data´s significance.

8. Groups vehicle-P21 and vehicle-adult have identical mean{plus minus}sem values for every gene analyzed. How is that possible? This casts a shadow of doubt over the entire data shown in Fig. 3.

9. Fig.5: why are these experiments only done during adulthood if the effect of perinatal fluoxetine on PNN expression was a lot more pronounced at P21? It would give a lot more information to be able to compare the data between P21 and adulthood.

10. Increase in fos positive neurons in adult hippocampus is interesting and essentially the only robust finding in the adult group. Were the fos positive neurons pyramidal cells or interneurons? This would be an important this to check. While no electrophysiology was done, changes in fos may provide indirect effects on whether the E/I balance in the adult hippocampus might be reduced (if interneurons fos+) or increased (if pyramids are fos+).

11. Fig. 5 H: The study focuses on interneurons, now morphology is show in pyramids, the authors should justify this switch in cell type. The "representative" figures are clearly different, this difference is not really reflected in the quantitative analysis. Perhaps there is a "more representative" images?

12. Some references are cited as referring to developmental effects of fluoxetine, whereas they actually describe FLX effects in adults. This is the case in the intro, first paragraph for Karpova 2011, and in the discussion for Kobayashi 2010. Authors need to be clear about this as the effects can be actually opposite in the perinatal and adult brain.

13. Reason for limiting study to males is unclear since behavioral effects PNFLX on anxiety and depression seem to equally affect male and females in most studies.

14. There is already a sizable literature on the effects of early life antidepressant treatment on various aspects of adult behavior and biochemistry. It is curious that while the authors study rats, they almost exclusively cite papers investigating mice and disregard the classical studies of Vogel et al on the long-term effects of clomipramine treatment. These should be cited.

15. The authors conclude that their data is consistent with disruption of hippocampal development, however, there is very little evidence of any disruption. Rather, these data suggest that fluoxetine directly inhibits PNN formation, which recovers soon after the cessation of the treatment. This would be consistent with the previous findings of Umemori and al., where a recovery of PNNs happened during the perinatal age, soon after the cessation of the treatment. Since the authors only study two time points, they must drastically tone down their interpretation of the data.

16. The authors seem to interpret that early reduction in PNNs has a causative effect on the (very limited) findings in the adult brain, but clarly the paper provides no evidence for such causative effects. Rather, these effects might be produced by the transient expression of SERT in hippocampal and cortical pyramidal neurons during the period of treatment, as shown in the classical studies of Gaspar and coworkers. These papers have not been cited or discussed at all.

17. Which part of the hippocampus was included in the analysis? Dorsal or ventral? As the authors probably know, many things are different between those two regions. The authors should always indicate whether they studied dorsal or the whole hippocampus.

18. Methods: the major findings of this manuscript are based on counting of neurons, but little details is provided as to how counting was done. To be reliable, cell countings should always be carried out using stereological techniques, but apparently they were not followed here. For example, it is questionable whether the very small difference in the CA1 PNNs in adults would survive a stereological counting.

19. For the adults, the rats were sacrificed on P80 histochemistry, P100 for westerns, and P120 for Golgi, why?

29. The authors suggest that PNFlx might have an impact on the closure of a critical period, but they have no evidence to support this notion. To begin with, what is the critical period for hippocampus, I do not think anybody knows. Please remove this part of discussion.

Minor issues:

• There are no page or line numbers and the figures have not been numbered, this complicates the review a lot.

• The authors correctly site Karpova 2011 on page 5, but on page 4, they probably mean Karpova 2009 that studied the effects of early fluoxetine treatment, in contrast to Karpova 2011 that only studied adults.

• Methods section lists solutions A, B and C for Golgi staining without any description of their contents or a reference. Please clarify.

In conclusion, the data provided is interesting although novelty is relatively limited. The results are clearly overinterpreted and they should be significantly toned down.
